# Cost-utility analysis and budget impact of policy options for kidney replacement therapy coverage for end-stage kidney disease in Kenya

**DOI:** 10.1136/bmjopen-2026-123693

**Published:** 2026-10-14

**Authors:** Patricia Nyokabi, Usa Chaikledkaew, Montarat Thavorncharoensap, Sitaporn Youngkong, Nyamai Mutono, Grace Ngaruiya, Ammarin Thakkinstian

**Affiliations:** 1 Mahidol University Health Technology Assessment (MUHTA) Graduate Program, Bangkok, Thailand; 2 Center for Epidemiological Modelling and Analysis, University of Nairobi, Nairobi, Kenya; 3 Social Administrative Pharmacy Division, Department of Pharmacy, Mahidol University, Faculty of Pharmacy, Bangkok, Thailand; 4 Global Health Technology Assessment (GHTA) Research Center, Faculty of Pharmacy, Mahidol University, Bangkok, Thailand; 5 Paul G Allen School for Global Animal Health, Washington State University, Pullman, Washington, USA; 6 School of Public Health, University of Nairobi, Nairobi, Kenya; 7 Department of Clinical Epidemiology and Biostatistics, Faculty of Medicine Ramathibodi Hospital, Mahidol University, Bangkok, Thailand

**Keywords:** Health policy, Health economics, HEALTH ECONOMICS, Nephrology

## Abstract

**Abstract:**

**Objectives:**

To evaluate the cost-utility and budget impact of alternative kidney replacement therapy (KRT) modalities and policy options for patients with end-stage kidney disease (ESKD) in Kenya.

**Design:**

Cost-utility and budget impact analyses were conducted using a decision-analytic Markov model. Deterministic one-way and probabilistic sensitivity analyses assessed parameter uncertainty. Model face validity was assessed through consultation with nephrology experts and external validation compared model-predicted survival among kidney transplant recipients with observed survival data. Budget impact was assessed over 5 years from the perspective of the Social Health Authority (SHA).

**Setting:**

Kenya

**Participants:**

Adults receiving KRT for ESKD.

**Interventions:**

Three KRT modalities, that is, peritoneal dialysis (PD), haemodialysis (HD) and kidney transplantation (KT) were compared. Compared with current practice (scenario 1), four policy scenarios were initiating all incident ESKD patients on PD (scenario 2), expanding KT coverage to achieve an additional 10% coverage within 5 years (scenario 3), redistributing coverage to 68% HD, 20% PD and 12% KT (scenario 4), and providing HD three times per week to 20% of patients currently receiving HD two times per week (scenario 5).

**Primary and secondary outcome measures:**

Primary outcomes were incremental cost-effectiveness ratio and incremental net monetary benefit. Secondary outcomes included costs, life years and quality-adjusted life years (QALYs) over 5-, 10-, 15-year and lifetime horizons.

**Results:**

PD had the highest lifetime costs compared with HD and KT. At a willingness-to-pay threshold of 120 000 Kenya shillings per QALY gained, KT was dominant compared with PD over the lifetime horizon and compared with HD over 10-year and 15-year horizons. Scenario 3 was dominant over 5-, 10- and 15-year horizons and had the lowest 5-year budget impact.

**Conclusions:**

Expanding access to KT appears economically efficient for improving ESKD coverage in Kenya, although decisions should consider uncertainty in key model parameters and health systems requirements for KT expansion.

**STRENGTHS AND LIMITATIONS OF THIS STUDY**

- Primary cost and utility data were collected from patients receiving haemodialysis and kidney transplantation, and a cohort-based Markov model was used to compare three kidney replacement therapy modalities and five policy scenarios.
- Multiple analytical time horizons (5, 10, 15 years and lifetime) were examined to assess the effect of time horizon on modelled costs and health outcomes.
- In the absence of a Kenyan kidney registry, transition probabilities were informed by the best available published evidence, although differences in clinical practice and patient characteristics may affect their applicability to Kenya.
- Cost and utility estimates for peritoneal dialysis were obtained from published evidence because of the limited availability of local data for this modality, introducing uncertainty into the model.
- Parameter uncertainty was explored using deterministic and probabilistic sensitivity analyses, and model validity was assessed through expert consultation and comparison with observed kidney transplantation survival data.

## Introduction

 End-stage kidney disease (ESKD) is characterised by an irreversible and progressive loss of kidney function to a level at which survival is not possible without kidney replacement therapy (KRT).^1 2^ Although KRT including haemodialysis (HD), peritoneal dialysis (PD) and kidney transplantation (KT) is life-sustaining and highly effective, access remains profoundly inequitable worldwide.^3^ It is estimated that up to 40% of individuals in high-income countries and as many as 96% in low-income countries (LICs), who require KRT are unable to access it.^4^

The economic burden of dialysis is substantial. The annual direct cost of dialysis per patient is equivalent to the average income of three individuals in upper-middle-income countries, 6 in low- and middle-income countries (LMICs) and 12 in LICs.^5^ Consequently, ESKD is a major contributor to catastrophic health expenditure and impoverishment globally.^6^ In sub-Saharan Africa, approximately 59% of patients discontinue dialysis despite ongoing clinical indication, primarily due to financial constraints.^7^ In South Africa, rationing policies were introduced to manage rising demand and limited supply of KRT, leading to only 30.7% of eligible patients accessing KRT between 2016 and 2018.^8^

Kenya, an LMIC with a population of approximately 53 million,^9^ has all three KRT modalities available. However, uptake of KT remains limited due to donor shortages, health system constraints and high costs.^10^ Between 2006 and 2021, only 466 kidney transplants were performed across both public and private facilities.^11^ HD is the predominant modality and has expanded rapidly following government investment, with dialysis units increasing from 17 in year 2000 to over 200 by 2017.^12^ Currently, an estimated 4000–5670 patients receive HD nationwide.^13 14^ In contrast, PD utilisation remains extremely low, with only 20–50 patients estimated to be on therapy.^15^

Kenya launched its Universal Health Coverage (UHC) programme in 2018. In 2024, reforms were undertaken by establishing a compulsory social health insurance model for all Kenyans, administered by the Social Health Authority (SHA). However, coverage of kidney disease services has posed major sustainability challenges. HD reimbursements always account for the top three of SHA’s expenditure.^16^ As a result of this and to manage costs, reimbursement rates for HD were reduced, prompting opposition from patients and healthcare professional associations.^16^ Currently, SHA reimburses up to two HD sessions per week at Kenya shillings (KSh) 10 650 per session.^17^ For KT, pre-transplant recipient evaluation is reimbursed up to KSh 150 000 per household annually, recipient surgery at KSh 700,000, donor nephrectomy at KSh 168,000, post-transplant care at KSh 200 000 annually per household. PD has been proposed for reimbursement at KSh 85 200 per month (up to 12 sessions monthly).^17^ Despite these provisions, both patients and providers continue to raise concerns regarding adequacy of benefits and reimbursement tariffs, respectively.

To date, no full economic evaluation comparing all three KRT modalities has been conducted in Kenya or elsewhere in Africa. Only one partial economic evaluation study, a cost analysis comparing HD and PD, was conducted in South Africa in 2019.^18^ As Kenya advances towards UHC, robust economic evidence is needed to guide priority setting and resource allocation. This study therefore aimed to assess the cost-utility and budget impact of KRT modalities and alternative coverage policy options to inform evidence-based decision making and optimal coverage for ESKD patients by the Ministry of Health and SHA in Kenya.

## Methods

### Study design

The cost-utility analysis was conducted from a societal perspective using a decision analytic Markov model. The target population was a hypothetical cohort of adult patients with ESKD entering the model at a mean age of 47.7 years, reflecting the average age of patients with ESKD observed in the Kenyan cross-sectional survey that informed the cost and utility estimates. This baseline age was selected to reflect the characteristics of the population represented in the underlying Kenyan data. Costs and health outcomes, including life years (LYs) and quality-adjusted life years (QALYs) were compared across all interventions.

### Intervention and comparator

Two analytical approaches were applied, that is, pairwise comparison and scenario analysis.

#### Pairwise comparison

HD, as the predominant modality in Kenya, was used as the comparator. PD and KT were evaluated as alternative interventions.

#### Scenario analysis

Five policy scenarios were evaluated. Scenario 1 (status quo) reflected the current distribution of KRT modalities (96% HD two times per week, 1% PD and 3% KT). Scenario 2 (PD-first) assumed that all incident ESKD patients were initiated on PD, except those with clinical contraindications. Scenario 3 (incremental KT expansion) modelled a progressive annual increase in KT to achieve an additional 10% coverage within 5 years (by 2028), with a corresponding reduction in HD, while maintaining PD at its current level. Scenario 4 (optimised modality mix) assumed a distribution of 12% KT, 20% PD and 68% HD, previously identified as the most cost-effective strategy.^19^ Scenario 5 (increased HD intensity) modelled HD three times per week for 20% of patients receiving HD, compared with the current practice of HD two times per week for all HD patients.

### Economic model

A cohort-based Markov model with four mutually exclusive health states including HD, PD, KT and death (absorbing state) was developed and is shown in figure 1.

**Figure 1:**
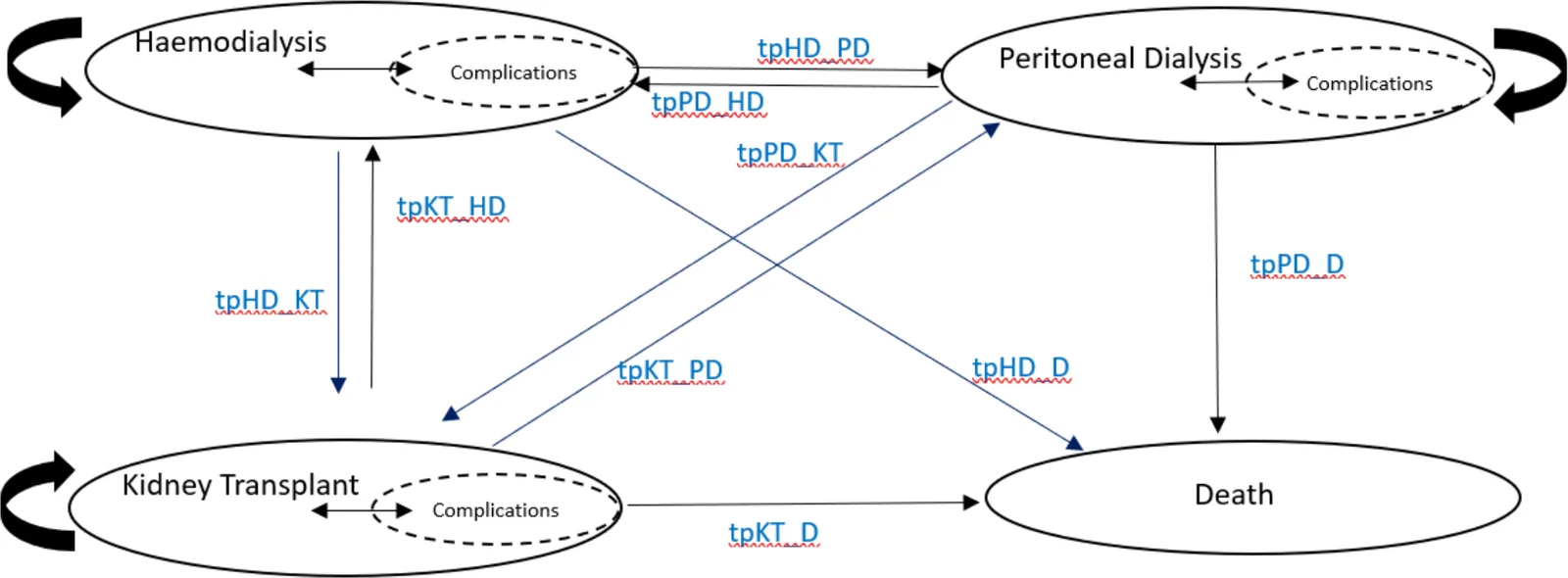
Schematic diagram of the Markov Model. HD, haemodialysis; KT, kidney transplantation; PD, peritoneal dialysis; tpHD_D, transition probability of moving from HD to death; tpHD_KT, transition probability of moving from HD to KT; tpHD_PD, transition probability of moving from HD to PD; tpKT_D, transition probability of moving from KT to death; tpKT_HD, transition probability of moving from KT to HD; tpKT_PD, transition probability of moving from KT to PD; tpPD_D, transition probability of moving from PD to death; tpPD_HD, transition probability of moving from PD to HD; tpPD_KT, transition probability of moving from PD to KT.

At model entry, patients were distributed across modalities according to proposed intervention or scenario under evaluation. During each annual cycle, patients could stay at their current state or transition between modalities or to death, based on transitional probabilities, derived from published literature and local data. The model incorporated modality-specific complications that could arise while patients were receiving any of the three KRT modalities. Patients with comorbidities were also accounted for within the model structure.

### Time horizon and discounting

The model was evaluated over 5-, 10-, 15-year and lifetime horizons. Both costs and health outcomes were discounted at an annual rate of 3%, consistent with recommendations from the WHO.^20^

### Model input parameters

Table 1 presents all input parameters used in this study. Input parameters included transition probabilities, costs and utility.

**Table 1:** Model input parameters

Parameter	Mean	SE	Distribution	95% CI		Source
				Lower	Upper	
Transition probabilities						
Probability of remaining on HD	0.6270	0.0627	Beta	0.5092	0.7406	
Probability of switching from HD to PD	0.0665	0.0067	Beta	0.0543	0.0807	^21^
Probability of switching from HD to KT	0.0035	0.0004	Beta	0.0029	0.0043	^21^
Probability of dying from HD	0.1350	0.0135	Beta	0.1097	0.1629	^21^
Probability of remaining on PD	0.5980	0.0598	Beta	0.4820	0.7125	
Probability of switching from PD to KT	0.0400	0.0040	Beta	0.0323	0.0480	^21^
Probability of switching from PD to HD	0.0750	0.0075	Beta	0.0610	0.0901	^21^
Probability of dying from PD	0.1250	0.0125	Beta	0.1032	0.1477	^21^
Probability of remaining on KT	0.7850	0.0785	Beta	0.6144	0.9175	
Probability of switching from KT to PD	0.0030	0.0003	Beta	0.0024	0.0036	^21^
Probability of switching from KT to HD	0.0350	0.0035	Beta	0.0290	0.0417	^21^
Probability of dying from KT	0.0170	0.0017	Beta	0.0136	0.0205	^21^
Probability of getting complications while on HD	0.1680	0.0168	Beta	0.1370	0.2031	^22^
Probability of getting complications while on PD	0.1620	0.0162	Beta	0.1326	0.1930	^22^
Probability of getting complications while on KT	0.1600	0.0160	Beta	0.1284	0.1962	^23^
Costs						
Total costs associated with HD in the first year	1 794 332	151 371	Gamma	1 510 096	2 098 142	Primary data collection
Total costs associated with PD in the first year	3 565 256	356 526	Gamma	2 901 029	4 320 437	Experts estimates
Total costs associated with KT in the first year	2 289 716	245 594	Gamma	1 841 385	2 825 957	Primary data collection
Total costs associated with HD from the second year onwards	1 754 702	147 408	Gamma	1 482 884	2 061 572	Primary data collection
Total costs associated with PD from the second year onwards	3 512 256	351 226	Gamma	2 864 339	4 273 835	Experts estimates
Total costs associated with KT from the second year onwards	853 908	108 439	Gamma	658 363	1 088 707	Primary data collection
Additional cost associated with third weekly session of HD	553 800	55 380	Gamma	444 178	661 875	Primary data collection
Cost associated with HD complications	41 656	4166	Gamma	34 386	50 308	^22^
Cost associated with PD complications	40 040	4004	Gamma	32 963	48 015	^24^
Cost associated with KT complications	130 000	13 000	Gamma	105 379	156 698	^25^
Utility						
Utility of HD patients	0.54	0.0579	Beta	0.43	0.66	Primary data collection
Utility of PD patients	0.61	0.0610	Beta	0.48	0.72	^19^
Utility of KT patients	0.86	0.0323	Beta	0.80	0.92	Primary data collection
Utility of HD patients with complications	0.44	0.0440	Beta	0.35	0.52	^22^
Utility of PD patients with complications	0.55	0.0550	Beta	0.44	0.65	^22^
Utility of KT patients with complications	0.56	0.0560	Beta	0.45	0.67	^26^

HD, haemodialysis; KT, kidney transplantation; PD, peritoneal dialysis.

#### Transition probabilities

Transition probabilities governing movement between health states for the ESKD cohort were derived from published studies.^19 21–26^ These probabilities informed annual transitions between HD, PD, KT and death within the Markov model.

#### Cost and utility data

Costs were estimated from a societal perspective and included direct medical, direct non-medical and indirect costs. Direct medical costs comprised consultation, diagnostic tests, hospital admission, medication, surgical procedures, palliative care and treatment monitoring, including laboratory and radiology services. Direct non-medical costs included patient and caregiver transportation, accommodation, meals and informal care. Indirect costs reflected productivity losses incurred by patients and caregivers due to time spent seeking, receiving or providing care and were calculated as the number of workdays lost multiplied by average daily wage.^27^ All costs were reported in 2024 KSh. In addition, health utilities were measured using the EuroQoL 5-Dimention 5-Level (EQ-5D-5L) instrument,^28^ a widely used preference-based measure for valuing health states and recommended by health technology assessment (HTA) agencies internationally.^29^ As a Kenya-specific value set was unavailable, the Ugandan value set was applied to derive utility values.^30^

Both cost and utility data were obtained from a cross-sectional study conducted between April and October 2024 at Kenyatta National Hospital (KNH), the largest teaching and referral hospital in Kenya. The target population comprised adult patients with ESKD receiving any of the three KRT modalities at KNH. Patients who had been receiving their current KRT modality for at least 3 months before data collection were eligible to participate. Patients who had discontinued treatment within the first 3 months of initiating KRT, switched from one KRT modality to another within 3 months before data collection, or had acute kidney injury were excluded. Patients who were mentally or physically unable to participate in the study were also considered ineligible. The 3-month criterion was applied to allow sufficient time for stabilisation following treatment initiation and to minimise the influence of early modality-related indications or complications.

Patients who met the inclusion criteria were identified during an initial assessment with nephrology nurses and were invited to participate in the study. After providing informed consent, eligible patients were interviewed face-to-face by a trained research assistant using a cost data collection form and the EQ-5D-5L questionnaire. HD patients were interviewed before undergoing their dialysis session for the day of data collection, whereas KT recipients were interviewed on their scheduled clinic day. The required sample size was calculated using the formula for estimating a population mean with a specified margin of error.^31^ The calculation used the expected mean utility values and SD reported in a previous study,^32^ with a two-sided significance level of 0.05 and an 18% relative margin of error. Based on these assumptions, the required sample size was 69 patients receiving HD and 50 KT recipients, resulting in a total target sample size of 119 patients. This sample size was considered sufficient to estimate the mean utility values for the HD and KT groups with the prespecified level of precision. All 119 eligible patients approached for participation agreed to participate and completed the interview, resulting in a 100% response rate. Thus, the planned sample size was fully achieved for both treatment groups. The number of patients receiving PD at KNH was insufficient to provide meaningful local estimates. Therefore, cost and utility estimates for PD were obtained from published literature.^19 22 24^

### Results presentation

Incremental cost-effectiveness ratio (ICER) and incremental net monetary benefit (INMB) values were estimated for all pairwise comparisons and policy scenarios. The ICER and INMB values were computed against the Kenyan willingness-to-pay (WTP) threshold of US$908 (equivalent to KSh 120 000) per QALY gained based on an opportunity cost-based threshold estimated by Kairu,^33^ informed by methodological approaches proposed by Woods *et al*^34^ and Ochalek *et al*.^35^

### Uncertainty analysis

To handle parameter uncertainty, both deterministic one-way and probabilistic sensitivity analyses (PSA) were performed. The PSA simultaneously varied all model parameters across predefined distributions and was iterated 1000 times. The results of the deterministic one-way sensitivity analysis were presented using tornado diagrams, while the PSA results were illustrated through cost-effectiveness planes and cost-effectiveness acceptability curves (CEAC).

### Model validation

The model underwent internal and external validation to enhance credibility and reduce structural and parameter uncertainty.^36^ Face validity was established through consultation with nephrology experts to confirm that the model structure, assumptions, inputs and results reflected clinical practice in the Kenyan context.^37^ External validation was performed by comparing model-predicted age-specific survival among KT recipients with observed survival data from a retrospective cohort study conducted at KNH by Correia,^38^ which examined clinical characteristics and outcomes of 125 KT patients between 2010 and 2019.

### Budget impact analysis

The total incremental budget for adopting and implementing the interventions under the scenario analysis was calculated over a 5-year period from the SHA’s perspective. In the absence of a Kenyan kidney registry, the incident and prevalent populations were estimated using data from the South Africa kidney registry in 2020^39^ and adjusted to reflect the Kenyan population size. Direct medical costs for each modality were subsequently obtained from primary data as well as supplemented with published studies.

## Results

### Cost-utility analysis

#### Pairwise comparison

Across all time horizons (5-, 10-, 15-year and lifetime), KT generated the highest LYs and QALYs followed by PD and HD (see online supplemental material). Over the 5-year horizon, the total cost for KT (KSh 6.19 million, 95% CIs: 5.36 to 7.08) was lower than that of HD (KSh 7.23 million (95% CI: 6.48 to 8.06) and PD (KSh 11.85 million, 95% CI: 10.20 to 13.54). KT was associated with higher QALYs than HD (2.72, 95% CI: 2.06 to 3.40) at lower cost (KSh 1.04 million, 95% CI: −2.12 to 0.08) and was therefore dominant (table 2). Similar findings were observed over the 10-year horizon, with KT dominating both HD and PD. Over the 15-year horizon, KT resulted in higher costs (KSh 0.81 million, 95% CI: −1.44 to 3.18) but substantially higher QALYs (7.94, 95% CI: 6.53 to 9.31), with an ICER of KSh 107 019 per QALY gained, below the WTP threshold of KSh 120 000 per QALY gained (table 2). Over the lifetime horizon, KT was dominant compared with PD, with lower cost (−KSh 0.61 million, 95% CI: −4.19 to 2.60) and higher QALYs (10.98, 95% CI: 9.02 to 13.01), and an INMB of KSh 1.93 million (95% CI, −1.28 to 5.45). In contrast, KT compared with HD over the lifetime horizon had an ICER of KSh 424 534 per QALY gained, exceeding the WTP threshold, with an INMB of −KSh 4.02 million (95% CI: −6.80 to −1.38). Overall, these findings indicate that the cost-effectiveness of KT relative to HD depends on the time horizon, whereas KT remained dominant compared with PD over the longer-term horizons.

**Table 2:** Cost-utility analysis results of three KRT modalities (pairwise comparisons)

Intervention vs comparator	Δ cost (million KSh)	Δ LYs	Δ QALYs	ICER (KSh per LY gained)	ICER (KSh per QALY gained)	INMB (million KSh)
5 year time horizon						
PD vs HD	4.62 (3.06, 6.30)	0.09 (−0.13, 0.29)	0.67 (0.02, 1.31)	52 227 388	6 954 096	−4.54 (−6.20, −3.02)
KT vs PD	−5.66 (−7.60, −3.91)	0.80 (0.65, 0.96)	2.06 (1.35, 2.70)	−7 074 610 *Dominant	−2 736 550 *Dominant	5.90 (4.19, 7.81)
KT vs HD	−1.04 (−2.12, 0.08)	0.88 (0.72, 1.06)	2.72 (2.06, 3.40)	−1 173 251 *Dominant	−381 503 *Dominant	1.36 (0.27, 2.43)
10-year time horizon						
PD vs HD	5.27 (3.37, 7.37)	0.29 (−0.15, 0.76)	1.08 (0.26, 1.91)	17 489 897	4 871 549	−5.14 (−7.22, −3.26)
KT vs PD	−5.88 (−8.75, −8.30)	2.36 (2.02, 2.75)	4.55 (3.48, 5.63)	−2 488 536 *Dominant*	−1 296 932 *Dominant	6.42 (3.85, 9.31)
KT vs HD	−0.61 (−2.47, 1.39)	2.65 (2.24, 3.02)	5.63 (4.58, 6.75)	−210 717 *Dominant	−99 896 *Dominant	1.29 (−0.58, 3.12)
15-year time horizon						
PD vs HD	5.50 (3.46, 7.86)	0.51 (−0.09, 1.15)	1.41 (0.42, 2.42)	10 656 347	3 913 588	−5.33 (−7.61, −3.34)
KT vs PD	−4.69 (−7.73, −1.92)	3.76 (3.20, 4.35)	6.54 (5.06, 7.93)	−1 251 235 *Dominant	−720 904 *Dominant	5.47 (2.78, 8.48)
KT vs HD	0.81 (−1.44, 3.18)	4.27 (3.68, 4.87)	7.94 (6.53, 9.31)	198 612	107 019	0.14 (−2.18, 2.36)
Lifetime horizon						
PD vs HD	6.21 (3.65, 9.06)	1.09 (0.25, 1.95)	2.20 (1.03, 3.46)	5 702 097	2 817 996	−5.95 (−8.67, −3.48)
KT vs PD	−0.61 (−4.19, 2.60)	7.07 (6.08, 8.04)	10.98 (9.02, 13.01)	−86 685 *Dominant	−55 794 *Dominant	1.93 (−1.28, 5.45)
KT vs HD	5.60 (2.93, 8.36)	8.16 (7.24, 9.07)	13.19 (11.20, 15.25)	686 208	424 534	−4.02 (−6.80, −1.38)

* Dominant means that the intervention is more effective and less costly than the comparator.

HD, haemodialysis; ICER, incremental cost-effectiveness ratio; INMB, incremental net monetary benefit; KRT, kidney replacement therapy; (KSh), Kenya shillings; KT, kidney transplantation; LYs, life years; PD, peritoneal dialysis; QALYs, quality adjusted life years.

#### Scenario analysis

Scenario 4 (12% KT, 20% PD, 68% HD) generated the highest LYs and QALYs across all time horizons, followed by scenario 3 (incremental KT expansion) and scenario 2 (PD-first) (online supplemental material). Scenarios 1 (status quo) and 5 (increased HD intensity) produced identical LYs and QALYs, indicating no additional health benefit from increasing HD frequency for 20% of patients under the model assumptions. Over a 5-year horizon, scenario 4 generated 4.13 QALYs (95% CI: 3.75 to 4.48) at a cost of KSh 8.01 million (95% CI: 7.27 to 8.80), compared with 3.75 QALYs (95% CI: 3.29 to 4.20) and KSh 7.23 million (95% CI: 6.44 to 8.10) for the status quo. Scenario 3 yielded 3.85 QALYs (95% CI: 3.41 to 4.28) at a lower cost of KSh 7.11 million (95% CI: 6.34 to 7.94), resulting in lower cost (−KSh 0.12 million; 95% CI: −0.18 to −0.07) and higher QALYs (0.10, 95% CI: 0.07 to 0.13) than the status quo and was therefore dominant (table 3). Similar results were observed at 10 and 15 years, with scenario 3 remaining dominant. At the lifetime horizon, scenario 3 generated 8.91 QALYs (95% CI: 7.76 to 10.19) at a cost of KSh 14.50 million (95% CI: 12.44 to 16.80), compared with 7.71 QALYs (95% CI: 6.56 to 8.98) and KSh 14.12 million (95% CI: 11.98 to 16.46) for the status quo, resulting in an ICER of KSh 314 186 per QALY gained, which exceeded the WTP threshold, and a negative INMB (−KSh 0.23 million, 95% CI: −0.52 to 0.02) (table 3). At a WTP threshold of KSh 120 000 per QALY gained, scenario 3 was cost-effective compared with the status quo over the 5-, 10- and 15-year horizons, whereas none of the alternative scenarios were cost-effective over the lifetime horizon.

**Table 3:** Cost-utility analysis results of KRT scenarios

KRT scenarios	Δ cost (million KSh)	Δ LYs	Δ QALYs	ICER (KSh per LY gained)	ICER (KSh per QALY gained)	INMB (million KSh)
5-year time horizon						
Scenario 1						
Scenario 2	0.40 (0.28, 0.54)	0.01 (−0.01, 0.03)	0.06 (0.00, 0.11)	52 227 388	6 954 096	−0.40 (−0.54, 0.27)
Scenario 3	−0.12 (−0.18, −0.07)	0.02 (0.02, 0.02)	0.10 (0.07, 0.13)	6 301 961 *Dominant	1 263 165 *Dominant	0.13 (0.08, 0.19)
Scenario 4	0.78 (0.46, 1.10)	0.10 (0.04, 0.15)	0.37 (0.22, 0.52)	8 097 705	2 105 870	−0.74 (−1.05, −0.42)
Scenario 5	0.34 (0.28, 0.42)	–	–	–	–	−0.34 (−0.42, −0.28)
10-year time horizon						
Scenario 1						
Scenario 2	0.47 (0.28, 0.67)	0.03 (−0.02, 0.07)	0.09 (0.02, 0.17)	17 489 897	4 871 549	−0.45 (−0.66, −0.27)
Scenario 3	−0.22 (−0.37, −0.07)	0.15 (0.12, 0.17)	0.36 (0.28, 0.44)	−1 483 779 *Dominant	−604 159 *Dominant	0.26 (0.11, 0.41)
Scenario 4	0.95 (0.45, 1.43)	0.29 (0.17, 0.41)	0.70 (0.48, 0.92)	3 223 217	1 344 889	−0.87 (−1.33, −0.38)
Scenario 5	0.45 (0.36, 0.56)	–	–	–	–	−0.45 (−0.56, −0.36)
15-year time horizon						
Scenario 1						
Scenario 2	0.50 (0.29, 0.70)	0.05 (−0.01, 0.11)	0.12 (0.04, 0.21)	10 756 067	3 972 190	−0.13 (−0.37, 0.12)
Scenario 3	−0.13 (−0.32, 0.09)	0.31 (0.27, 0.35)	0.61 (0.50, 0.72)	−415 719 *Dominant	−209 974 *Dominant	1.93 (1.57, 2.27)
Scenario 4	1.15 (0.64, 1.65)	0.49 (0.33, 0.65)	0.99 (0.73, 1.25)	2 358 350	1 159 128	1.77 (1.02, 2.52)
Scenario 5	0.49 (0.38, 0.61)	–	–	–	–	−0.49 (−0.61, −0.38)
Lifetime horizon						
Scenario 1						
Scenario 2	0.55 (0.33, 0.78)	0.10 (0.02–0.17)	0.19 (0.09, 0.30)	5 742 186	2 885 222	−0.53 (−0.75, −0.32)
Scenario 3	0.38 (0.11–0.66)	0.74 (0.66–0.83)	1.20 (1.02, 1.38)	507 676	314 186	−0.23 (−0.52, 0.02)
Scenario 4	1.69 (1.15, 2.28)	0.94 (0.73–1.14)	1.60 (1.29, 1.93)	1 800 540	1 061 162	−1.50 (−2.08, 0.95)
Scenario 5	0.52 (0.41, 0.65)	–	–	–	–	−0.52 (−0.65, 0.41)

* Dominant means that the intervention is more effective and less costly than the comparator.

ICER, incremental cost-effectiveness ratio; INMB, incremental net monetary benefit; KRT, kidney replacement therapy; (KSh), Kenya shillings; LYs, life years; QALYs, quality adjusted life years.

### Uncertainty analysis

#### One-way sensitivity analysis

One-way analysis results are shown in supplement material. In the pairwise analysis of KT compared with PD during the lifetime horizon, among all varied input parameters, the ICER results were most sensitive to total costs associated with PD in the first year and from the second year onwards, and to discounting rate for outcomes and costs. In scenario analysis of scenario 3 compared with scenario 1, in the 10-year time horizon, ICER results were most sensitive to discounting rate for outcomes and for costs and total costs associated with PD and then HD and then KT from the second year onwards. The results for the most cost-effective pair and scenario analyses are presented herein, while all other results are reported in the supplementary materials.

#### Probabilistic sensitivity analysis

For the pairwise comparisons, the CEAC indicated that at a WTP threshold of KSh 120 000 per QALY gained, HD had a 100% probability of being the most cost-effective intervention over the lifetime horizon (figure 2). In the scenario analysis, scenario 1 had a 98% probability of being the most cost-effective strategy for the scenario analysis over the lifetime horizon (figure 3). The cost-effectiveness planes generated from 1000 iterations of the PSA are provided in the supplementary material.

**Figure 2:**
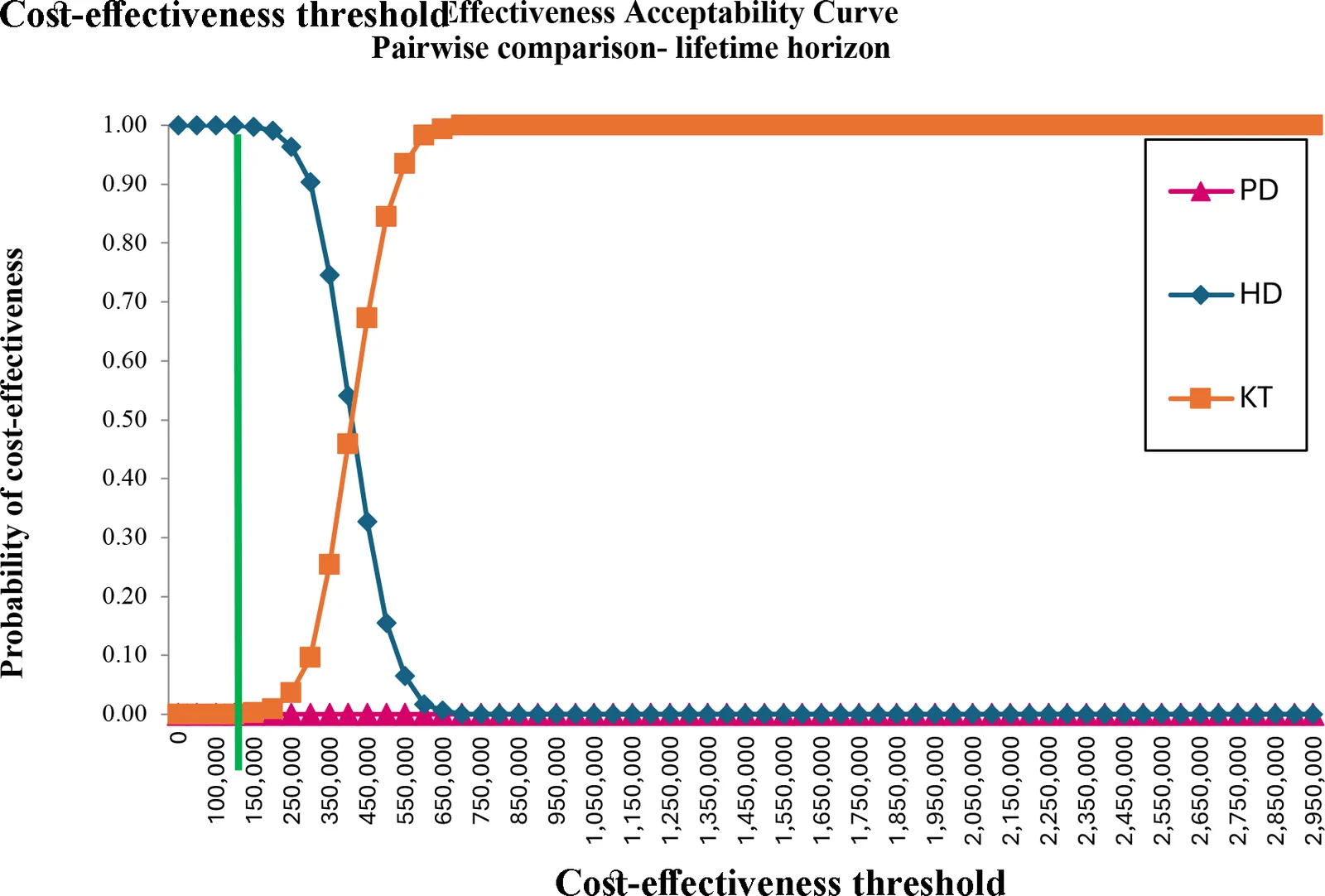
Cost-effectiveness acceptability curves for pairwise comparison over the lifetime horizon. HD, haemodialysis; KT, kidney transplantation; PD, peritoneal dialysis; QALY, quality-adjusted life year.

**Figure 3:**
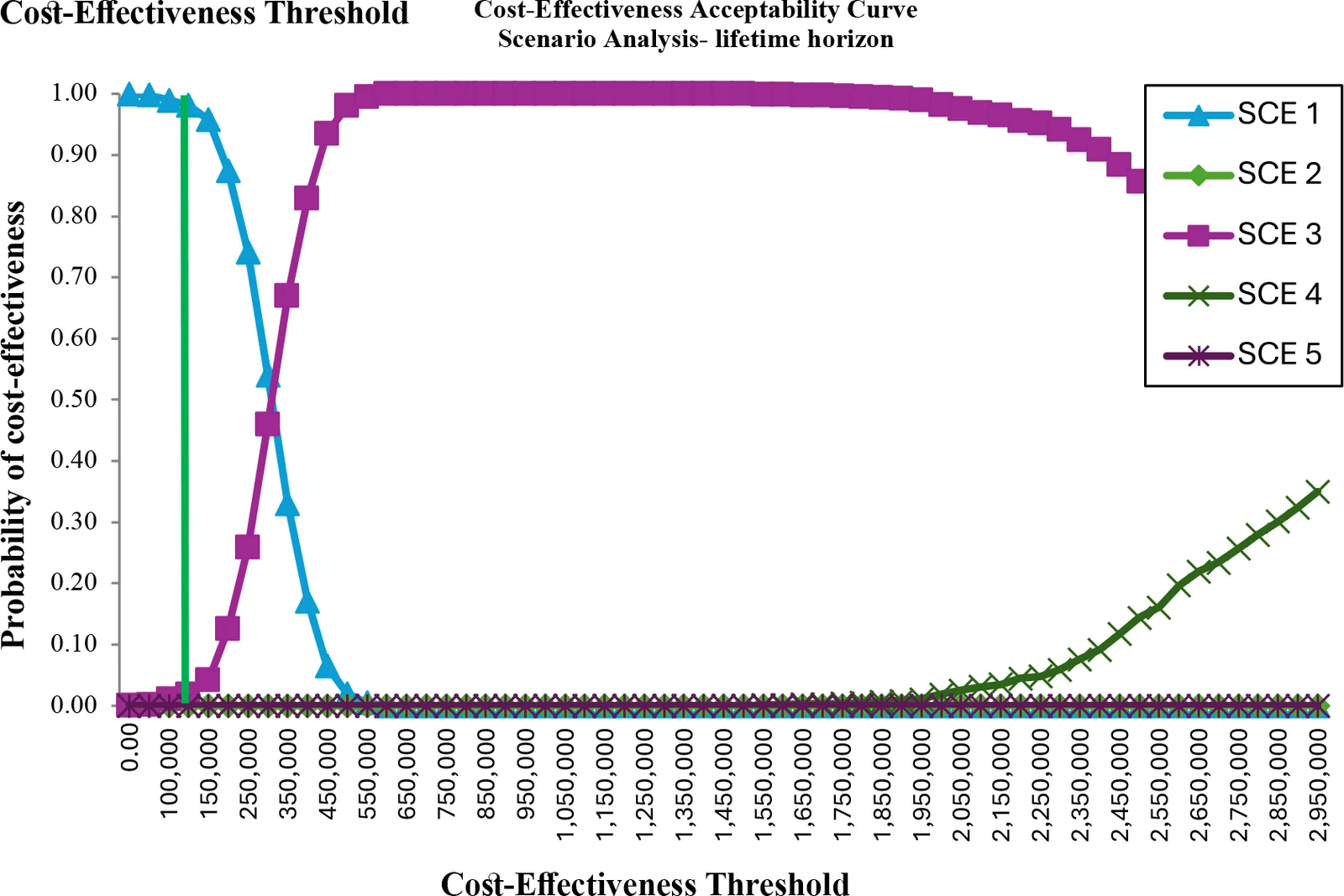
Cost-effectiveness acceptability curves for scenario analysis over the lifetime horizon. QALY, quality-adjusted life year; SCE, scenario.

### Model validation

Results of the external validation are presented in figure 4. The modelled cumulative age-specific survival probabilities from 0 to 132 months (11 years) following KT demonstrated a declining survival trend over time, consistent with empirical survival data from a recent Kenyan cohort study. During the early post-transplant period (0–48 months), the modelled survival curve closely approximated observed real-world data, indicating strong external validity of short- to mid-term survival estimates. However, beyond this period, modelled survival probabilities were progressively lower than those observed empirically, suggesting a potential underestimation of long-term survival among KT recipients. To address this discrepancy, the model-predicted allograft survival was externally calibrated to published survival estimates reported by Correia *et al*.^38^

**Figure 4:**
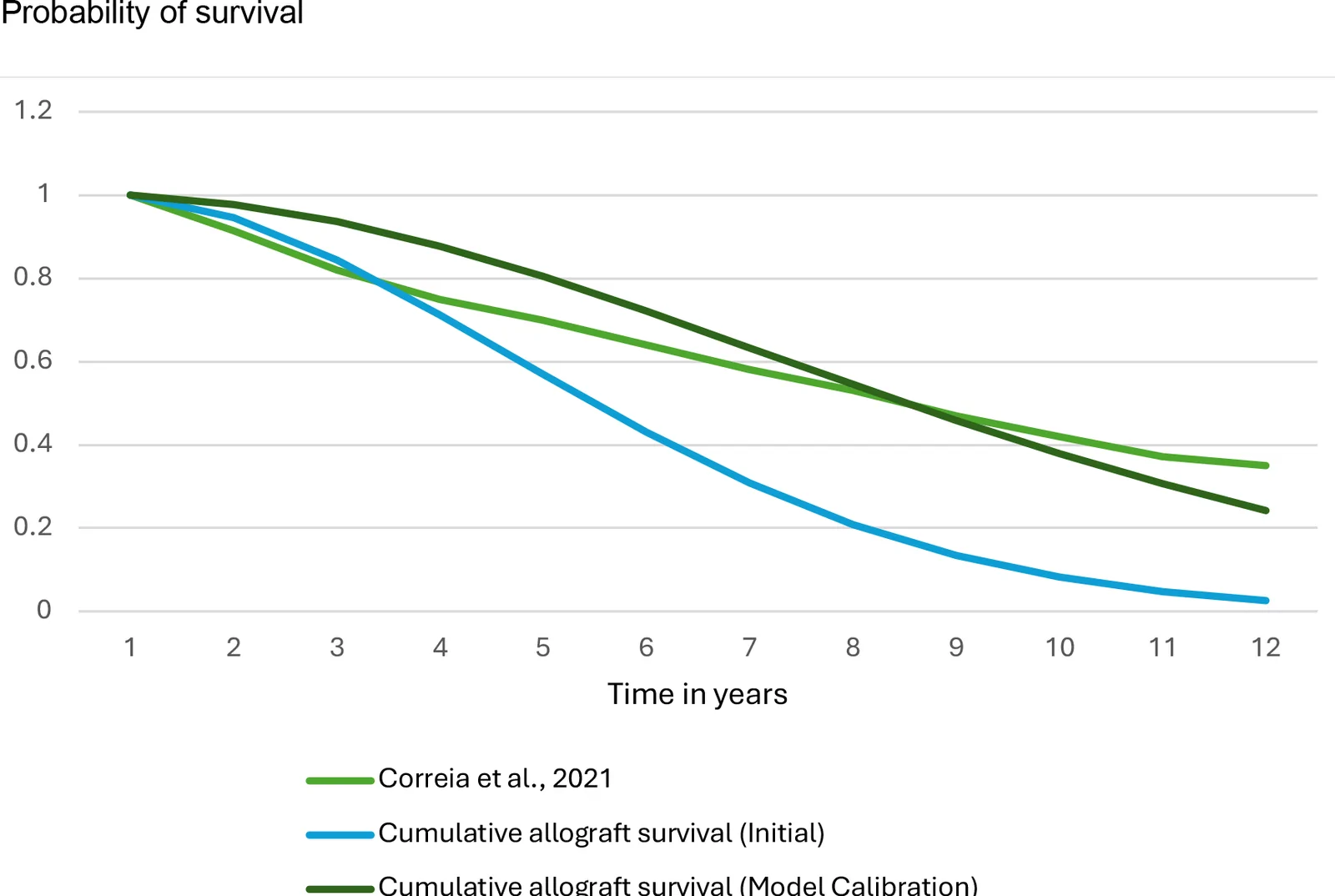
Model validation results.

### Budget-impact analysis

The estimated number of prevalent ESKD cases was 8,199, with 722 incident cases annually. Over the 5-year projection period, the total cumulative cost under scenario 1 (KSh 46.2405 billion) and scenario 3 (KSh 46.2402 billion) were almost similar with an incremental difference of only KSh 0.0002 billion (table 4). These findings indicate that expanding KT coverage would be essentially cost-neutral relative to the status quo. Importantly, KT (KSh 42.85 billion) was considerably less costly than PD (KSh 123.91 billion) over 5 years. The incremental budget attributable to PD reached KSh 81.07 billion, underscoring the considerable long-term savings potential associated with shifting the treatment modality mix towards KT.

**Table 4:** Budget impact for the scenario analysis in billion (KSh)

Year	Total budget		Incremental budget	Total budget		Incremental budget
	KT	PD	PD	Scenario 1	Scenario 3	Scenario 3
1	17.42	31.63	14.20	8.4577	8.4577	0.00000
2	5.67	26.97	21.30	8.8462	8.8462	(0.00002)
3	6.14	23.87	17.73	9.4355	9.4354	(0.00004)
4	6.59	21.57	14.98	9.7116	9.7115	(0.00007)
5	7.02	19.87	12.85	9.7896	9.7894	(0.00011)
**Total**	42.85	123.91	81.07	46.2405	46.2402	(0.0002)

KSh, Kenya shillings; KT, kidney transplantation; PD, peritoneal dialysis.

## Discussion

This study evaluated the cost-utility of KRT for ESKD in Kenya from a societal perspective. Across all time horizons, KT generated the highest LYs and QALYs, followed by PD and HD. These findings align with prior systematic reviews demonstrating the survival and quality-of-life advantages of transplantation over dialysis.^19 40^ KT more closely restores kidney function than dialysis and is associated with improved physical functioning, psychosocial well-being and greater opportunities for occupational reintegration.^41^

PD incurred the highest total costs across all time horizons. Although PD is often less costly than HD in high-income settings, evidence from LMICs suggests that limited economies of scale, reliance on imported consumables and low utilisation rates can reverse this relationship.^42 43^ Kenya’s cost structure may therefore reflect the broader challenges affecting KRT delivery in sub-Saharan Africa, underscoring the importance of local market conditions, procurement arrangements and health system capacity when assessing the cost-effectiveness of dialysis modalities.^44^

The results also demonstrated that the time horizon substantially influenced cost comparisons. At the 5- and 10- year horizons, KT was less costly than HD. However, over the lifetime horizon, HD appeared less costly than KT, partly due to the shorter survival associated with HD and thus lower cumulative treatment costs. This finding is consistent with evidence from a systematic review^42^ showing substantially higher cumulative mortality among patients receiving HD than among those receiving KT. These results highlight an important consideration in chronic disease modelling. Interventions that extend survival may generate greater cumulative healthcare costs over the lifetime, while simultaneously producing substantially greater health gains. Consequently, lifetime cost differences should be interpreted alongside QALYs and the resulting cost-effectiveness estimates rather than in isolation.

The scenario analysis further demonstrated that modest, sustained increases in KT coverage could provide substantial economic value. A gradual annual increase in KT to achieve an additional 10% coverage over 5 years accompanied by a corresponding reduction in HD was the only scenario that remained cost-effective compared with the status quo. This finding is consistent with evidence from Australia,^45^ where incremental expansion of transplantation capacity was also shown to improve economic efficiency. Expanding KT coverage increases accumulated QALYs while reducing long-term dependence on dialysis, potentially improving health outcomes while reducing healthcare expenditures over the longer term. The INMB results supported KT as the most cost-effective option compared with PD and under the incremental KT expansion scenario over the relevant time horizons. This is consistent with findings from a systematic review in which KT generated a positive INMB in 12 of 13 studies.^19^ Collectively, these findings suggest that gradual expansion of KT capacity may be a more feasible and economically attractive approach than abrupt redistribution of KRT provision in Kenya and other LMICs.

The uncertainty analyses suggested that discount rate had the greatest influence on ICER estimates, reflecting the long-term accumulation of costs and health outcomes in ESKD. This highlights the importance of applying discounting assumptions that are consistent with international HTA guidance. The ICERs were also sensitive to annual costs of PD, both in the first year and from the second year onward, indicating that assumptions about PD delivery costs were important drivers of the model results. Despite this sensitivity, PSA showed that the relative ranking of modalities remained stable, supporting the robustness of the overall conclusions.

From a budgetary perspective, scenarios involving substantial expansion of PD generated the greatest budget impact, reflecting its relatively high cost profile in the Kenyan setting. In contrast, scenarios prioritising KT expansion, particularly gradual increases in KT coverage, produced the lowest budget impact and were associated with cost savings. For countries pursuing UHC, these findings suggest that improving clinical outcomes while maintaining fiscal sustainability does not necessarily have to compete with these objectives. However, the feasibility of expanding KT depends on the availability of transplantation infrastructure, specialist workforce, organ donation systems and sustainable financing mechanisms.

This study strengthens the evidence base by combining primary utility and cost data with modelling of pairwise comparisons across three KRT modalities and realistic policy scenarios. Evaluating multiple time horizons (5, 10, 15 years and lifetime) was particularly important, given the chronic nature of ESKD and the potential for economic conclusions to vary according to the analytic horizon. However, several limitations should be considered. First, the modelled cohort was hypothetical, with a baseline mean age of 47.7 years derived from the Kenyan cross-sectional survey that informed the cost and utility estimates. Although this age reflects the characteristics of the population represented in the underlying Kenyan data, the cohort may not fully represent the broader population of patients with ESKD in Kenya. Differences in patient characteristics and the distribution of KRT modalities across the wider Kenyan population may therefore affect the generalisability of the model results. Second, in the absence of a national kidney registry, transition probabilities were obtained from published studies conducted in Iran, Thailand and Mexico,^21–23^ and regional epidemiological data were adapted to the Kenyan context.^39^ Differences in disease progression, mortality, treatment patterns and healthcare delivery across these settings may limit the generalisability of these parameters to Kenya. Third, PD utility values were obtained from published studies rather than primary Kenyan data because the number of patients receiving PD in Kenya was too small to provide reliable local estimates. The use of non-Kenyan utility data for PD patients may limit the applicability of these utility estimates to the Kenyan context and may introduce uncertainty into comparisons involving PD. Last, uncertainty surrounding the costs of PD is an important limitation. In particular, fluctuations in importation and procurement prices could materially affect the cost-effectiveness of PD and its relative prioritisation against other KRT modalities. Nevertheless, the sensitivity analyses indicated that the main conclusions remained robust across the ranges tested. As local data become available, periodic reassessment of the cost-effectiveness and budget impact of KRT modalities will be important to reduce uncertainty and inform decisions regarding PD scale-up and reimbursement.

Beyond efficiency considerations, these findings also have important equity implications. Access to KT in many LMICs remains constrained by limited infrastructure, workforce capacity, organ donation systems and financing mechanisms. These constraints contribute to heavy reliance on facility-based HD, which may be geographically concentrated and less accessible to rural and socioeconomically disadvantaged populations. Strategic expansion of KT capacity, together with the development of deceased donor programmes and equitable referral pathways could improve access to transplantation while reducing the longer-term financial burden associated with dialysis. Incorporating transplantation into national benefit packages may therefore contribute to both economic efficiency and more equitable access to KRT. For countries seeking to achieve UHC under constrained fiscal conditions, aligning financing reforms with investments in transplantation infrastructure, organ donation systems and patient education could generate sustained population health gains while potentially reducing catastrophic health expenditure. Strengthening transplantation systems should therefore be considered not only as a clinical intervention but also as part of broader health-system reform for chronic disease care in sub-Saharan Africa.

## Conclusions

KT was the most cost-effective modality compared with PD over the lifetime horizon and cost-effective compared with HD over 5-, 10- and 15-year horizons at a WTP threshold of KSh 120 000 per QALY gained. A gradual annual increase in KT coverage to achieve an additional 10% within 5 years, with a corresponding reduction in HD, emerged as the most economically efficient and fiscally sustainable policy option and was associated with the lowest budget impact. For Kenya and similar LMIC settings, policies that progressively expand transplantation capacity through investment in deceased donor programmes, workforce development, infrastructure and public awareness could deliver substantial gains in survival and quality of life while supporting more sustainable KRT financing. Further research and periodic reassessment using local data will be important as transplantation capacity and KRT delivery systems evolve.

## Supplementary material

10.1136/bmjopen-2026-123693 online supplemental file 1

## Data Availability

All data relevant to the study are included in the article or uploaded as supplementary information.
