# Supplementary material for: Cost-utility analysis and budget impact of policy options for kidney replacement therapy coverage for end-stage kidney disease in Kenya
Source: BMJ Open. 2026 Oct 14;16(10):e123693. doi: 10.1136/bmjopen-2026-123693 (PMC13691753; doi:10.1136/bmjopen-2026-123693)
Supplement: Supplementary file 1 [file bmjopen-2026-123693.reviewer_comments.pdf]

# PEER REVIEW HISTORY

BMJ Open publishes all reviews undertaken for accepted manuscripts. Reviewers are asked to complete a checklist review form and are provided with free text boxes to elaborate on their assessment. These free text comments are reproduced below.

## ARTICLE DETAILS

### Title (Provisional)

Cost-Utility Analysis and Budget Impact of Policy Options for Kidney Replacement Therapy Coverage for End-stage Kidney Disease in Kenya

### Authors

Nyokabi, Patricia; Chaikledkaew, Usa; Thavorncharoensap, Montarat; Youngkong, Sitaporn; Mutono, Nyamai; Ngaruiya, Grace; Thakkinstian, Ammarin

---

## VERSION 1 - REVIEW

---

| | |
|---|---|
| **Reviewer** | **1** |
| **Name** | **Kelson, Zoe** |
| **Affiliation** | **University of Exeter, Mathematics** |
| **Date** | **13-Aug-2026** |
| **COI** | **None** |

---

This study evaluated the cost -utility and budget impact of alternative KRT modalities and policy options to support evidence -informed decision making in Kenya.

Reviewer comments:

"A decision -analytic Markov model was used to compare the cost -utility of KRT modalities as well as four policy scenarios against the status quo" [Abstract]

The analytical approach taken is succinctly described by the authors in the Abstract.

"Uncertainty analyses, model validation and budget -impact analysis were performed. " [Abstract]

Can the authors please add more detail regarding these analyses in the Abstract?

"The cost -utility analysis was conducted from a societal perspective using a decision analyticMarkov model...

A cohort-based Markov model with four mutually exclusive health states was developed: HD, PD, KT, and death (absorbing state ), as shown in Figure 1."

An appropriate modelling methodolgy has been applied by the authors.

Can the full equations and model formulation please be detailed in the supplementary material?

"The target population was a hypothetical cohort of adult patients with ESKD entering the model at a mean age of 47.7 years, reflecting the average age for ESKD patients in Kenya"

Can the authors please clarify whether the hypothetical cohort can be considered to be representative?

"Tables 1 presents all input paraments used in this study. Input parameters included transition probabilities, costs and utility."

The authors do well to tabulate the transition rates with source (citation) and uncertainty (distribution).

"Costs and health outcomes, including life years (LYs) and quality adjusted life years (QALYs) were compared across all interventions...

HD, as the predominant modality in Kenya, was used as the comparator. PD and KT were evaluated as alternative interventions"

A valuable pair-wise analysis has been conducted by the authors.

"The alternative scenarios included: initiating all incident ESKD patients on PD (scenario 2 ), annual incremental expansion of KT to achieve an additional 10% coverage over five years (scenario 3 ), redistribution of coverage to HD 68 %, PD 20% and KT 12% (scenario 4 ), and provision of thrice -weekly HD for 20% of patients currently receiving twice weekly HD, (scenario 5). " [Abstract]

and

"Five policy scenarios were evaluated. Scenario 1 (Status quo) reflected the current distribution of KRT modalities (96% HD twice weekly, 1 % PD and 3% KT). Scenario 2 (PD - first) assumed that all incident ESKD patients were initiated on PD, except those with clinical contraindications. Scenario 3 (Incremental KT expansion) modelled a progressive annual increase in KT to achieve an additional 10 % coverage within five years (by 2028 ), with a corresponding reduction in HD, while maintaining PD at current level. Scenario 4 (Optimized modality mix) assumed a distribution of 12% KT, 20% PD and 68% HD ), previously identified as the most cost -effective strategy (19). Scenario 5 (Increased HD intensity) modelled three - times -weekly HD for 20% patients receiving HD, compared with the current practice of twice -weekly HD for all HD patients. "

A number of insightful what-if scenarios have been explored by the authors.

"The incremental cost -effectiveness ratios (ICERs) and incremental net benefits (INBs) were estimated for all pairwise comparisons and policy scenarios. ICERs and INBs were computed against the Kenyan willingness -to -pay (WTP ) threshold of US$908 per QALY gained (equivalent to KShs 120,000 ), based on an opportunity cost -based threshold estimated by Kairu (31 ), informed by methodological approaches proposed by Woods (32 ) and Ochalek (33). "

Suitable model outcomes have been reported by the authors.

"To handle parameter uncertainty, both deterministic one -way and probabilistic sensitivity analyses (PSA) were performed. The PSA simultaneously varied all model parameters across predefined distributions and was iterated 1000 times. The results of the deterministic one - way sensitivity analysis were presented using tornado diagrams, while the PSA results were illustrated through cost -effectiveness acceptability curves."

The authors have rigorously quantified and explored uncertainty in their model outcomes.

"Face validity was established through consultation with nephrology experts to confirm that the model structure, assumptions, inputs and results reflected clinical practice in Kenyan context (35). External validation was performed by comparing model -predicted age -specific survival among KT recipients with observed survival data from a retrospective cohort study conducted at KNH by Correia (36), which examined clinical characteristics and outcomes of 125 KT patients between 2010 and 2019. "

The authors do well to undertake model verification and validation.

"At the WTP threshold of KShs 120,000 per QALY, KT was cost -effective compared with PD over the lifetime horizon (ICER =KShs 55,794 per QALY gained). KT was also cost -effective compared with HD at the 10 - year (ICER =KShs 99,896 per QALY gained) and 15 -year (ICER =KShs 107,019 per QALY gained) horizons. "

and

"Table 2: Cost-utility analysis of three KRT modalities (pairwise comparisons)"

and

"Table 3: Cost-utility analysis of five KRT scenarios "

Can confidence intervals please be generated and reported throughout the Results text?

"Over the 5 -year horizon, KT dominated HD, as it generated higher QALYs at lower cost. However, over the lifetime horizon, although HD was less costly than KT, it produced substantially fewer QALYs, resulting in an ICER that exceeded the WTP threshold"

Can model outcomes (i.e. values with CIs) please be reported alongside study findings throughout the Results text?

Furthermore, can the authors please consider statistically comparing model outcomes for each intervention and scenario?

Can the PSA outcomes please also be shown in a scatter plot with WTP threshold?

"The analysis relied on published transition probabilities and regional epidemiological estimates because Kenya does not have a national renal registry, which may limit the precision of long -term projections. • Peritoneal dialysis cost and utility estimates were obtained from published literature owing to the very low number of patients receiving this modality in Kenya, which may affect the generalisability of the model inputs." [Strengths and Limitations of the study]

and

"However, limitations include reliance on published transition probabilities 66 (21 -23) due to the absence of a national renal registry, and adaptation of regional epidemiological data (37). 67 These gaps highlight the urgent need for investment in renal surveillance systems to support evidence - 68 informed policy"

The authors have provided a Discussion on the study limitations.

Thanks to the authors for providing a copy of the CHEERS checklist.

---

| | |
|---|---|
| **Reviewer** | **2** |
| **Name** | **Luo, Xuyang** |
| **Affiliation** | **University of Electronic Science and Technology of China Sichuan Provincial People's Hospital, Department of Nephrology** |
| **Date** | **15-Aug-2026** |
| **COI** | **None** |

---

The literature search strategy was systematic and comprehensive, conducting a cost-utility and budget impact analysis using a decision-analytic Markov model, based on the actual payment pressures faced by Kenya's Social Health Insurance (SHA) system, evaluating three renal replacement therapies (HD, PD, KT) under five policy scenarios. The budget impact analysis (over 5 years), framed from SHA's payer perspective, offers direct reference value for policymakers.

It is recommended that the uncertainty surrounding PD cost estimates and its implications for policy recommendations be more thoroughly discussed. The utility values used in QALY calculations may contain potential biases. It should be clearly stated whether the utility values for PD are derived from Kenyan population data; if not, this limitation should be explicitly highlighted in the discussion, along with an assessment of its potential impact on the conclusions. The transition probabilities in the model lack local Kenyan data support and are entirely sourced from international literature, including studies from Iran, Thailand, and Mexico. This limitation must be clearly acknowledged in the discussion.

---

## VERSION 1 - AUTHOR RESPONSE

**Reviewer comments**
**Reviewer 1:**

**Comments to the Author**
This study evaluated the cost -utility and budget impact of alternative KRT modalities and policy options to support evidence -informed decision making in Kenya.

**Response:** We sincerely thank Professor Zoe Kelson for positive assessment of the modelling approach and for the constructive suggestions to further improve the methodological transparency and reporting of the manuscript.

1. "A decision-analytic Markov model was used to compare the cost -utility of KRT modalities as well as four policy scenarios against the status quo." [Abstract] The analytical approach taken is succinctly described by the authors in the Abstract. "Uncertainty analyses, model validation and budget -impact analysis were performed. " [Abstract] Can the authors please add more detail regarding these analyses in the Abstract?

**Response:** Thank you very much for this helpful suggestion. We have expanded the description of the analytical methods in the Abstract as follows (Abstract, lines 26-31, page 2). The revised Abstract now provides additional information on the deterministic and probabilistic sensitivity analyses, model validation, and the five-year budget impact analysis, while maintaining the required concise format.

*"Design: Cost-utility and budget impact analyses were conducted using a decision-analytic Markov model. Deterministic one-way and probabilistic sensitivity analyses assessed parameter uncertainty. Model face validity was assessed through consultation with nephology experts and external validation compared model-predicted survival among kidney transplant recipients with observed survival data. Budget impact was assessed over five years from the perspective of the Social Health Authority (SHA).*

2. "The cost -utility analysis was conducted from a societal perspective using a decision analytic Markov model...A cohort-based Markov model with four mutually exclusive health states was developed: HD, PD, KT, and death (absorbing state), as shown in Figure 1." An appropriate modelling methodolgy has been applied by the authors. Can the full equations and model formulation please be detailed in the supplementary material?

**Response:** We thank the reviewer for this valuable suggestion. We have provided the full mathematical formulation of the cohort-based Markov model in the supplementary material (page 1-2), including the model structure, transition probabilities, cost and utility calculations, and the calculation of incremental costs, QALYs, and economic outcomes. This additional information is intended to improve the transparency and reproducibility of the model.

3. "The target population was a hypothetical cohort of adult patients with ESKD entering the model at a mean age of 47.7 years, reflecting the average age for ESKD patients in Kenya" Can the authors please clarify whether the hypothetical cohort can be considered to be representative?

**Response:** Thank you for raising this important point. The hypothetical cohort was parameterised using the mean age of 47.7 years observed in the Kenyan cross-sectional survey that informed the cost and utility estimates. Thus, the baseline age was intended to reflect the characteristics of the population represented in the underlying Kenyan data. We have clarified this rationale in the Methods and acknowledged in the Discussion that, although the baseline age reflects the underlying survey population, the hypothetical cohort may not fully represent the broader population of patients with ESKD in Kenya.

(Methods, lines 118-122, page 7)

*"The target population was a hypothetical cohort of adult patients with ESKD entering the model at a mean age of 47.7 years, reflecting the average age of patients with ESKD observed in the Kenyan cross-sectional survey that informed the cost and utility estimates.*

*This baseline age was selected to reflect the characteristics of the population represented in the underlying Kenyan data."*

(Discussion, lines 365-371, page 18)

*"First, the modelled cohort was hypothetical, with a baseline mean age of 47.7 years derived from the Kenyan cross-sectional survey that informed the cost and utility estimates. Although this age reflects the characteristics of the population represented in the underlying Kenyan data, the cohort may not fully represent the broader population of patients with ESKD in Kenya. Differences in patient characteristics and the distribution of KRT modalities across the wider Kenyan population may therefore affect the generalisability of the model results."*

4. "Tables 1 presents all input paraments used in this study. Input parameters included transition probabilities, costs and utility." The authors do well to tabulate the transition rates with source (citation) and uncertainty (distribution).
**Response:** We sincerely thank the reviewer for this positive assessment of the presentation of the model input parameters.

5. "Costs and health outcomes, including life years (LYs) and quality adjusted life years (QALYs) were compared across all interventions...HD, as the predominant modality in Kenya, was used as the comparator. PD and KT were evaluated as alternative interventions" A valuable pair-wise analysis has been conducted by the authors.

**Response:** We sincerely thank the reviewer for this positive assessment of the pairwise cost-utility analyses.

6. "The alternative scenarios included: initiating all incident ESKD patients on PD (scenario 2), annual incremental expansion of KT to achieve an additional 10% coverage over five years (scenario 3), redistribution of coverage to HD 68 %, PD 20% and KT 12% (scenario 4), and provision of thrice -weekly HD for 20% of patients currently receiving twice weekly HD, (scenario 5)." [Abstract] "Five policy scenarios were evaluated. Scenario 1 (Status quo) reflected the current distribution of KRT modalities (96% HD twice weekly, 1 % PD and 3% KT). Scenario 2 (PD -first) assumed that all incident ESKD patients were initiated on PD, except those with clinical contraindications. Scenario 3 (Incremental KT expansion) modelled a progressive annual increase in KT to achieve an additional 10 % coverage within five years (by 2028), with a corresponding reduction in HD, while maintaining PD at current level. Scenario 4 (Optimized modality mix) assumed a distribution of 12% KT, 20% PD and 68% HD), previously identified as the most cost -effective strategy (19). Scenario 5 (Increased HD intensity) modelled three -times -weekly HD for 20% patients receiving HD, compared with the current practice of twice -weekly HD for all HD patients. " A number of insightful what-if scenarios have been explored by the authors.

**Response:** We sincerely thank the reviewer for this positive assessment of the policy scenario analyses. We aimed to explore alternative KRT configurations that could be relevant to healthcare resource allocation and policy planning in Kenya.

7. "The incremental cost -effectiveness ratios (ICERs) and incremental net benefits (INBs) were estimated for all pairwise comparisons and policy scenarios. ICERs and INBs were computed against the Kenyan willingness -to -pay (WTP) threshold of US$908 per QALY gained (equivalent to KShs 120,000), based on an opportunity cost -based threshold estimated by Kairu (31), informed by methodological approaches proposed by Woods (32 ) and Ochalek (33). " Suitable model outcomes have been reported by the authors.

**Response:** We sincerely thank the reviewer for this positive assessment of the reported model

outcomes.

8. "To handle parameter uncertainty, both deterministic one -way and probabilistic sensitivity analyses (PSA) were performed. The PSA simultaneously varied all model parameters across predefined distributions and was iterated 1000 times. The results of the deterministic one - way sensitivity analysis were presented using tornado diagrams, while the PSA results were illustrated through cost -effectiveness acceptability curves." The authors have rigorously quantified and explored uncertainty in their model outcomes.

**Response:** We sincerely thank the reviewer for this positive assessment. We have retained the deterministic and probabilistic sensitivity analyses and have further clarified their methods and presentation in the revised manuscript and supplementary material.

9. "Face validity was established through consultation with nephrology experts to confirm that the model structure, assumptions, inputs and results reflected clinical practice in Kenyan context (35). External validation was performed by comparing model -predicted age -specific survival among KT recipients with observed survival data from a retrospective cohort study conducted at KNH by Correia (36), which examined clinical characteristics and outcomes of 125 KT patients between 2010 and 2019. " The authors do well to undertake model verification and validation.

**Response:** We sincerely thank the reviewer for this positive assessment. We have retained the description of face validity and external validation in the revised manuscript and have provided additional methodological details to improve transparency.

10. "At the WTP threshold of KShs 120,000 per QALY, KT was cost -effective compared with PD over the lifetime horizon (ICER =KShs 55,794 per QALY gained). KT was also cost -effective compared with HD at the 10 - year (ICER =KShs 99,896 per QALY gained) and 15 -year (ICER =KShs 107,019 per QALY gained) horizons. " and "Table 2: Cost-utility analysis of three KRT modalities (pairwise comparisons)" and "Table 3: Cost-utility analysis of five KRT scenarios " Can **confidence intervals** please be generated and reported throughout the Results text?

**Response:** Thank you very much for this important suggestion. We agree that reporting uncertainty around the model outcomes improves the interpretation of the results. As the model outcomes including total costs, total LYs, total QALYs, incremental costs, incremental LYs, incremental QALYs, and incremental net monetary benefit (INMB) were evaluated using probabilistic sensitivity analysis (PSA), we have reported the corresponding 95% confidence intervals (CI) in the revised Tables 2 and 3, based on the 2.5th and 97.5th percentiles of the PSA simulations.

We have not reported 95% CI for the ICERs. The ICER is a ratio of incremental costs to incremental QALYs, and its sampling distribution can be highly skewed or unstable when incremental QALYs are close to zero or cross zero. Consequently, a simple percentile-based interval for the ICER may be difficult to interpret and may not adequately represent uncertainty around the cost-effectiveness ratio. Previous methodological research has highlighted these limitations and recommended approaches that account for the two-dimensional nature of uncertainty in incremental costs and effects (1, 2). Therefore, rather than reporting potentially misleading ICER intervals, we present uncertainty in the cost-

effectiveness results using the distribution of incremental costs and incremental QALYs from the PSA in cost-effectiveness planes and cost-effectiveness acceptability curves. This approach provides a more appropriate representation of uncertainty surrounding the cost-effectiveness conclusions.

**References**


1. Briggs AH, Mooney CZ, Wonderling DE. Constructing confidence intervals for cost-effectiveness ratios: an evaluation of parametric and non-parametric techniques using Monte Carlo simulation. Stat Med. 1999;18(23):3245–3262. doi:10.1002/(SICI)1097-0258(19991215)18:23<3245::AID-SIM314>3.0.CO;2-2.
2. Heitjan DF, Moskowitz AJ, Whang W. Problems with interval estimates of the incremental cost-effectiveness ratio. Med Decis Making. 1999;19(1):9–14. doi:10.1177/0272989X9901900102.


11. "Over the 5 -year horizon, KT dominated HD, as it generated higher QALYs at lower cost. However, over the lifetime horizon, although HD was less costly than KT, it produced substantially fewer QALYs, resulting in an ICER that exceeded the WTP threshold"
Can **model outcomes (i.e. values with CIs)** please be reported alongside study findings throughout the Results text?

**Response:** Thank you for this helpful suggestion. We have revised the cost-utility results section to provide uncertainty estimates alongside the principal model outcomes where appropriate. The reported estimates are accompanied by the corresponding 95% confidence intervals derived from the probabilistic sensitivity analysis as follows (Results, lines 228-264, page 12-13).

***"Pairwise comparison***

*Across all time horizons (5-, 10-, 15-year, and lifetime), KT generated the highest LYs and QALYs followed by PD and HD (see supplement material). Over the 5-year horizon, the total cost for KT (KSh 6.19 million, 95% confidence intervals (CI): 5.36–7.08) was lower than that of KT (KSh 7.23 million (95% CI: 6.48–8.06) for HD and PD (KSh 11.85 million, 95% CI: 10.20–13.54). KT was associated with higher QALYs than HD (2.72, 95% CI: 2.06–3.40) at lower cost (KSh 1.04 million, 95% CI: −2.12-0.08) and was therefore dominant. Similar findings were observed over the 10-year horizon, with KT dominating both HD and PD. Over 15-year horizon, KT resulted in higher costs (KSh 0.81 million, 95% CI: −1.44 to 3.18) but substantially higher QALYs (7.94, 95% CI: 6.53–9.31), with an ICER of KSh 107,019 per QALY gained, below the WTP threshold of KSh 120,000 per QALY gained (Table 2). Over the lifetime horizon, KT was dominant compared with PD, with lower cost (−KSh 0.61 million, 95% CI: −4.19 to 2.60) and higher QALYs (10.98, 95% CI: 9.02–13.01), and an INMB of KSh 1.93 million (95% CI, −1.28 to 5.45). In contrast, KT compared with HD over the lifetime horizon had an ICER of KSh 424,534 per QALY gained, exceeding the WTP threshold, with an INMB of −KSh 4.02 million (95% CI: −6.80 to −1.38). Overall, these findings indicate that the cost-effectiveness of KT relative to HD depends on the time horizon, whereas KT remained dominant compared with PD over the longer-term horizons.*

***Scenario analysis***

*Scenario 4 (12% KT, 20% PD, 68% HD) generated the highest LYs and QALYs across all time horizons, followed by scenario 3 (incremental KT expansion) and scenario 2 (PD-first) (Supplement material). Scenarios 1 (status quo) and 5 (increased HD intensity) produced identical LYs and QALYs, indicating no additional health benefit from increasing HD frequency for 20% of patients under the model assumptions. Over a 5-year horizon, scenario 4 generated 4.13 QALYs (95% CI: 3.75–4.48) at a cost of KSh 8.01 million (95% CI: 7.27–8.80), compared with 3.75 QALYs (95% CI: 3.29–4.20) and KSh 7.23 million (95% CI: 6.44–8.10) for the status quo. Scenario 3 yielded 3.85 QALYs (95% CI: 3.41–4.28) at a lower cost of KSh 7.11 million (95% CI: 6.34–7.94), resulting in lower cost (−Ksh 0.12 million; 95% CI: −0.18 to −0.07) and higher QALYs (0.10, 95% CI: 0.07–0.13) than the status quo and was therefore dominant (Table 3). Similar results were observed at 10 and 15 years, with scenario 3 remaining dominant. At the lifetime horizon, scenario 3 generated 8.91 QALYs (95% CI: 7.76–10.19) at a cost of KSh 14.50 million (95% CI: 12.44–16.80), compared with 7.71 QALYs (95% CI: 6.56–8.98) and KSh 14.12 million (95% CI: 11.98–16.46) for the status quo, resulting in an ICER of KSh 314,186 per QALY gained, which exceeded the WTP threshold, and a negative INMB (-KSh 0.23 million, 95% CI: −0.52 to 0.02). At a WTP threshold of KSh 120,000 per QALY gained, scenario 3 was cost-effective compared with the status quo over the 5-, 10-, and 15-year horizons, whereas none of the alternative scenarios were cost-effective over the lifetime horizon."*

12. Furthermore, can the authors please consider **statistically comparing model outcomes** for each intervention and scenario?

**Response:** We thank the reviewer for this valuable suggestion. We considered statistically comparing model outcomes across the three KRT interventions and the five policy scenarios. However, conventional statistical hypothesis testing, such as t-tests, is not generally the primary approach for comparing outcomes generated by decision-analytic cost-effectiveness models. Economic evaluations are primarily concerned with characterising parameter uncertainty and decision uncertainty and assessing how this uncertainty affects cost-effectiveness decisions, rather than testing whether model-generated costs, LYs, or QALYs are statistically significantly different between alternatives. This is particularly relevant because model outcomes are generated from multiple input parameters and structural assumptions rather than from independent samples of observed individuals (1).

Furthermore, ICERs are ratio measures and can have highly skewed or unstable distributions, particularly when incremental QALYs are small or approach zero. Consequently, conventional statistical hypothesis tests and confidence intervals may be difficult to interpret for cost-effectiveness ratios (2). Accordingly, rather than applying conventional t-tests to the model outputs, we assessed uncertainty using 1,000 probabilistic sensitivity analysis (PSA) simulations. We report 95% confidence intervals for total costs, total LYs, total QALYs, and incremental costs, incremental QALYs, and incremental net monetary benefit (INMB), and present the uncertainty in incremental costs and QALYs using cost-effectiveness planes. We also report the probability that each intervention or scenario is cost-effective at the specified willingness-to-pay threshold using cost-effectiveness acceptability curves. These analyses provide a decision-relevant assessment of uncertainty and allow the extent of overlap and variation in model outcomes across alternatives to be evaluated without relying on potentially

inappropriate hypothesis testing. This approach is consistent with established good-practice recommendations for economic modelling (1).


**References**

1. Briggs AH, Weinstein MC, Fenwick EAL, Karnon J, Sculpher MJ, Paltiel AD. Model parameter estimation and uncertainty: a report of the ISPOR-SMDM Modeling Good Research Practices Task Force Working Group–6. Med Decis Making. 2012;32(5):722–732. doi:10.1177/0272989X12458348.
2. Briggs AH, Fenn P. Confidence intervals or surfaces? Uncertainty on the cost-effectiveness plane. Health Econ. 1998;7(8):723–740. doi:10.1002/(SICI)1098-1053(199812)7:8<723::AID-HEC392>3.0.CO;2-O.


13. Can the PSA outcomes please also be shown in a scatter plot with WTP threshold?

**Response:** This was indeed an oversight. Thank you very much to the reviewer for highlighting this. The cost-effectiveness planes generated from 1,000 iterations of the PSA are provided in the supplementary material (Figures 6-7, page 7).

14. "The analysis relied on published transition probabilities and regional epidemiological estimates because Kenya does not have a national renal registry, which may limit the precision of long -term projections. • Peritoneal dialysis cost and utility estimates were obtained from published literature owing to the very low number of patients receiving this modality in Kenya, which may affect the generalisability of the model inputs." [Strengths and Limitations of the study] and "However, limitations include reliance on published transition probabilities 66 (21 -23) due to the absence of a national renal registry, and adaptation of regional epidemiological data (37). 67 These gaps highlight the urgent need for investment in renal surveillance systems to support evidence - 68 informed policy" The authors have provided a Discussion on the study limitations.

**Response:** We sincerely thank the reviewer for this positive assessment. We have retained the discussion of the key methodological limitations and have further strengthened the discussion of uncertainty surrounding the model inputs, particularly the use of published transition probabilities, regional epidemiological estimates, and non-Kenyan utility estimates for PD.

15. Thanks to the authors for providing a copy of the CHEERS checklist.

**Response:** We sincerely thank the reviewer for acknowledging the provision of the CHEERS checklist.

**Reviewer 2:**

**Comments to the Author**

The literature search strategy was systematic and comprehensive, conducting a cost-utility and budget impact analysis using a decision-analytic Markov model, based on the actual payment pressures faced by Kenya's Social Health Insurance (SHA) system, evaluating three

renal replacement therapies (HD, PD, KT) under five policy scenarios. The budget impact analysis (over 5 years), framed from SHA's payer perspective, offers direct reference value for policymakers.

**Response:** We sincerely thank Dr. Xuyang Luo for the positive assessment of the study and for the constructive comments regarding uncertainty in the model inputs and their implications for policy interpretation.

1. It is recommended that the uncertainty surrounding PD cost estimates and its implications for policy recommendations be more thoroughly discussed.

**Response:** We thank the reviewer for highlighting this important issue. We have expanded the limitations and discussion sections to provide a more detailed consideration of uncertainty surrounding the PD cost estimates. In particular, we now discuss the limited availability of Kenya-specific PD cost data and the potential implications of this uncertainty for the relative cost-effectiveness of PD compared with HD and KT. We have also considered this uncertainty in the sensitivity analyses and have clarified that policy conclusions regarding the optimal KRT modality mix should be interpreted in light of the uncertainty surrounding the PD cost estimates. Please see the revised text below (Discussion, lines 379-382, page 19)

*"Last, uncertainty surrounding the costs of PD is an important limitation. In particular, fluctuations in importation and procurement prices could materially affect the cost-effectiveness of PD and its relative prioritization against other KRT modalities."*

2. The utility values used in QALY calculations may contain potential biases. It should be clearly stated whether the utility values for PD are derived from Kenyan population data; if not, this limitation should be explicitly highlighted in the discussion, along with an assessment of its potential impact on the conclusions.

**Response:** We thank the reviewer for raising this important point. The utility value for PD was not derived from a Kenyan population. This was primarily because the number of patients receiving PD in Kenya is very small and no published Kenya-specific utility data for patients receiving PD were available. We have now made this limitation explicit in the revised manuscript. We have also assessed the potential impact of uncertainty in the PD utility estimate through probabilistic sensitivity analysis. The revised Discussion explains that the use of non-Kenyan utility data may limit the applicability of the estimate to the Kenyan context and may introduce uncertainty into comparisons involving PD. Please see the revised text as follows.

(Discussion, lines 375-377, page 18)

*"Third, PD utility values were obtained from published studies rather than primary Kenyan data because the number of patients receiving PD in Kenya was too small to provide reliable local estimates. The use of non-Kenyan utility data for PD patients may limit the applicability of these utility estimates to the Kenyan context and may introduce uncertainty into comparisons involving PD."*

3. The transition probabilities in the model lack local Kenyan data support and are entirely sourced from international literature, including studies from Iran, Thailand, and Mexico. This limitation must be clearly acknowledged in the discussion.

**Response:** Thank you very much for this important observation. We agree that the lack of Kenya-specific transition probabilities is an important limitation of the model. We have explicitly acknowledged this limitation in the revised Discussion. Because Kenya does not currently have a comprehensive national renal registry providing modality-specific longitudinal transition data, transition probabilities were obtained from available international evidence. We have clarified that differences in patient characteristics, healthcare delivery, clinical practice, and survival across settings may affect the applicability of these estimates to the Kenyan context. The uncertainty associated with these parameters was incorporated into the probabilistic sensitivity analysis, but we acknowledge that uncertainty analysis cannot fully compensate for the absence of high-quality local longitudinal data. We therefore recommend that future Kenyan renal registry and longitudinal cohort data be used to update and refine the model when such evidence becomes available. Please see the revised text as follows (Discussion, lines 371-375, page 18).

*"Second, in the absence of national kidney registry, transition probabilities were obtained published studies conducted in Iran, Thailand and Mexico (21-23), and regional epidemiological data were adapted to Kenyan context (39). Differences in disease progression, mortality, treatment patterns, and healthcare delivery across these settings may limit the generalisability of these parameters to Kenya."*
