# Supplementary material for: Cost-utility analysis and budget impact of policy options for kidney replacement therapy coverage for end-stage kidney disease in Kenya
Source: BMJ Open. 2026 Oct 14;16(10):e123693. doi: 10.1136/bmjopen-2026-123693 (PMC13691753; doi:10.1136/bmjopen-2026-123693)
Supplement: Supplementary file 2 [file bmjopen-16-10-s001.docx]

**Supplementary Material**

A. **Schematic diagram of Markov model, equation, and formulation**

Figure 1: Schematic diagram of the Markov Model

**Transition probabilities**

The model is a discrete-time cohort Markov model with a cycle length of 1 year. The transition probability matrix is:

$$P=\left( \begin{matrix} tp_{\mathrm{HD}\_\mathrm{HD}} & tp_{\mathrm{HD}\_\mathrm{PD}} & tp_{\mathrm{HD}\_\mathrm{KT}} & tp_{\mathrm{HD}\_D} \\ tp_{\mathrm{PD}\_\mathrm{HD}} & tp_{\mathrm{PD}\_\mathrm{PD}} & tp_{\mathrm{PD}\_\mathrm{KT}} & tp_{\mathrm{PD}\_D} \\ tp_{\mathrm{KT}\_\mathrm{HD}} & tp_{\mathrm{KT}\_\mathrm{PD}} & tp_{\mathrm{KT}\_\mathrm{KT}} & tp_{\mathrm{KT}\_D} \\ 0 & 0 & 0 & 1 \end{matrix} \right)$$

Each row sums to 1, i.e. $\sum_{j\in S} tp_{i\_j}=1$ for all $i\in${HD,PD,KT,D}.

The "remain in state" probabilities (diagonal terms, shown as self-loops in Figure 1) were calculated by subtraction:

$tp_{\mathrm{HD}\_\mathrm{HD}}=1-tp_{\mathrm{HD}\_\mathrm{PD}}-tp_{\mathrm{HD}\_\mathrm{KT}}-tp_{\mathrm{HD}\_D}$

$$tp_{\mathrm{PD}\_\mathrm{PD}}=1-tp_{\mathrm{PD}\_\mathrm{HD}}-tp_{\mathrm{PD}\_\mathrm{KT}}-tp_{\mathrm{PD}\_D}$$

$tp_{\mathrm{KT}\_\mathrm{KT}}=1-tp_{\mathrm{KT}\_\mathrm{HD}}-tp_{\mathrm{KT}\_\mathrm{PD}}-tp_{\mathrm{KT}\_D}$

Death is an absorbing state ($tp_{D\_D}$=1).

All transitional probabilities are outlined in Table 1 in the main text.

**Complications**

Complications were modelled as an expected-value adjustment applied within each parent state per cycle, using state-specific complication probabilities $p_{i}^{\mathrm{comp}}$:

$$C_{i}^{\mathrm{adj}}=C_{i}+p_{i}^{\mathrm{comp}}\cdot C_{i}^{\mathrm{comp}}$$

$U_{i}^{\mathrm{adj}}=U_{i}-p_{i}^{\mathrm{comp}}\cdot D_{i}^{\mathrm{comp}}$

where $C_{i}^{\mathrm{comp}}$ and $D_{i}^{\mathrm{comp}}$ are the cost and utility decrement associated with a complication event in state $i$. Complications therefore affect the costs and QALYs accrued while a patient remains in a given state, but do not alter the underlying transition probabilities between HD, PD, KT and D.

B. **Results of total costs and health outcomes**

Table 1: Total costs and health outcomes of three KRT modalities (pairwise comparisons)

| KRT modality | **Total Cost**  **(Million Ksh)** | **Total LYs** | **Total QALYs** |
| --- | --- | --- | --- |
| **5-year time horizon** | | | |
| HD | 7.23  (6.48, 8.06) | 3.64  (3.47, 3.81) | 3.66  (3.18, 4.16) |
| PD | 11.85  (10.20, 13.54) | 3.73  (3.58, 3.88) | 4.33  (3.85, 4.84) |
| KT | 6.19  (5.36, 7.08) | 4.53  (4.49, 4.55) | 6.38  (5.83, 6.88) |
| **10-year time horizon** | | | |
| HD | 10.73  (9.38, 12.30) | 5.23  (4.82, 5.67) | 5.35  (4.58, 6.08) |
| PD | 16.00  (13.72, 18.78) | 5.52  (5.14, 5.87) | 6.44  (5.71, 7.22) |
| KT | 10.12  (8.76, 11.63) | 7.88  (7.76, 7.99) | 10.98  (10.03, 11.91) |
| **15-year time horizon** | | | |
| HD | 12.34  (10.52, 14.50) | 5.98  (5.33, 6.65) | 6.20  (5.30, 7.17) |
| PD | 17.85  (15.16, 20.68) | 6.49  (5.90, 7.06) | 7.60  (6.68, 8.62) |
| KT | 13.16  (11.43, 15.03) | 10.24  (9.99, 10.51) | 14.14  (12.84, 15.32) |
| **Lifetime horizon** | | | |
| HD | 13.83  (11.68, 16.12) | 6.85  (5.94, 7.74) | 7.29  (6.10, 8.52) |
| PD | 20.04  (17.13, 23.60) | 7.94  (7.04, 8.87) | 9.50  (8.19, 10.93) |
| KT | 19.43  (16.75, 22.31) | 15.01  (14.14, 15.89) | 20.48  (18.45, 22.64) |

*Dominant means that the intervention is more effective and less costly than the comparator. ICER, Incremental Cost-Effectiveness Ratio; HD, Haemodialysis; KRT, Kidney Replacement Therapy; KT, Kidney Transplant; LYs, Life Years; PD, Peritoneal Dialysis; QALYs, Quality Adjusted Life Years; INMB, Incremental Net monetary benefit; Confidence Intervals, CI.

Table 2: Total costs and health outcomes of five KRT scenarios

| **KRT Modality** | **Total costs (Million Ksh)** | **Total LYs** | **Total QALYs** |
| --- | --- | --- | --- |
| **5-year time horizon** | | | |
| Scenario 1 | 7.23 (6.44, 8.10) | 3.67 (3.50, 3.84) | 3.75 (3.29, 4.20) |
| Scenario 2 | 7.64 (6.85, 8.50) | 3.68 (3.52, 3.83) | 3.81 (3.37, 4.23) |
| Scenario 3 | 7.11 (6.34, 7.94) | 3.69 (3.52, 3.85) | 3.85 (3.41, 4.28) |
| Scenario 4 | 8.01 (7.27, 8.80) | 3.77 (3.64, 3.89) | 4.13 (3.75, 4.48) |
| Scenario 5 | 7.58 (6.77, 8.46) | 3.67 (3.50, 3.84) | 3.75 (3.29, 4.20) |
| **10-year time horizon** | | | |
| Scenario 1 | 10.74 (9.53, 12.10) | 5.30 (4.90, 5.73) | 5.53 (4.81, 6.23) |
| Scenario 2 | 11.21 (9.99, 12.51) | 5.33 (4.94, 5.71) | 5.62 (4.94, 5.72) |
| Scenario 3 | 1052 (9.38, 11.77) | 5.45 (5.07, 5.86) | 5.89 (5.20, 6.57) |
| Scenario 4 | 11.69 (10.53, 12.95) | 5.60 (5.27, 5.62) | 6.23 (5.62, 6.83) |
| Scenario 5 | 11.20 (9.94, 12.54) | 5.31 (4.60, 5.73) | 5.53 (4.81, 6.23) |
| **15-year time horizon** | | | |
| Scenario 1 | 12.35 (10.70, 14.19) | 6.09 (5.51, 6.68) | 6.44 (5.58, 7.35) |
| Scenario 2 | 12.85 (11.20, 14.64) | 6.13 (5.84, 6.65) | 6.56 (5.75, 7.43) |
| Scenario 3 | 12.22 (10.65, 13.93) | 6.40 (5.84, 6.95) | 7.05 (6.25, 7.89) |
| Scenario 4 | 13.50 (11.94, 15.16) | 6.57 (6.09, 7.07) | 7.43 (6.67, 8.20) |
| Scenario 5 | 12.84 (11.12, 14.73) | 6.09 (5.51, 6.68) | 6.44 (5.58, 7.35) |
| **Lifetime horizon** | | | |
| Scenario 1 | 14.12 (11.98, 16.46) | 7.11 (6.30, 8.01) | 7.71 (6.56, 8.98) |
| Scenario 2 | 14.68 (12.54, 17.03) | 7.21 (6.41, 8.10) | 7.90 (6.78, 9.14) |
| Scenario 3 | 14.50 (12.44, 16.80) | 7.85 (7.04, 8.72) | 8.91 (7.76, 10.19) |
| Scenario 4 | 15.82 (13.70, 18.10) | 8.05 (7.31, 8.86) | 9.31 (8.19, 10.49) |
| Scenario 5 | 14.65 (12.44, 17.00) | 7.11 (6.30, 8.01) | 7.71 (6.55, 8.98) |

*Dominant means that the intervention is more effective and less costly than the comparator. ICER, Incremental Cost-Effectiveness Ratio; HD, Haemodialysis; KRT, Kidney Replacement Therapy; KT, Kidney Transplant; LYs, Life Years; PD, Peritoneal Dialysis; QALYs, Quality Adjusted Life Years; INMB, Incremental Net monetary benefit; Confidence Intervals, CI.

C. **Results of one-way sensitivity analysis**

Figure 2: Tornado diagram for KT compared with PD during a lifetime horizon

Tornado diagram Pairwise analysis HD-PD

|  | Lower bound values | Upper bound values |
| --- | --- | --- |
| Discounting rate for outcomes | -124.908% | -110.74% |
| Transition probability from Haemodialysis to Death | -122.371% | -113.453% |
| Discounting rate for costs | -118.922% | -110.949% |
| Total Costs associated with Peritoneal Dialysis from the second year onwards | -120.873% | -114.063% |
| Transition probability from Kidney Transplant to Death | -119.042% | -115.699% |
| Transition probability from Haemodialysis to Peritoneal Dialysis | -118.207% | -116.49% |
| Transition probability from Kidney Transplant to Haemodialysis | -118.105% | -116.429% |
| Total Costs associated with Haemodialysis in the first year | -118.171% | -116.557% |
| Total Costs associated with Haemodialysis from the second year onwards | -118.061% | -116.495% |
| Transition probability from Peritoneal Dialysis to Death | -117.829% | -116.843% |

Figure 3: Tornado diagram for Pairwise analysis between HD and PD

Tornado diagram Pairwise analysis HD-KT

|  | Lower bound values | Upper bound values |
| --- | --- | --- |
| Discounting rate for costs | -50.9485% | 118.122% |
| Discounting rate for outcomes | -37.9563% | 43.6408% |
| Transition probability from Haemodialysis to Death | -16.2457% | 13.3999% |
| Total Costs associated with Haemodialysis in the first year | -5.76916% | 6.29403% |
| Total Costs associated with Haemodialysis from the second year onwards | -15.0053% | -3.05589% |
| Transition probability from Kidney Transplant to Death | -5.97899% | 5.3886% |
| Total Costs associated with Kidney Transplant from the second year onwards | 14.6349% | 25.9337% |
| Transition probability from Peritoneal Dialysis to Death | -3.74415% | 4.19576% |
| Transition probability from Haemodialysis to Peritoneal Dialysis | -3.2109% | 3.49634% |
| Transition probability from Kidney Transplant to Haemodialysis | -2.10089% | 2.53756% |

Figure 4: Tornado diagram for Pairwise analysis between HD and KT

Tornado diagram Societal perspective SCE 1- SCE 2

|  | Lower bound values | Upper bound values |
| --- | --- | --- |
| Discounting rate for outcomes | -77.0792% | -48.1595% |
| Discounting rate for costs | -71.8532% | -45.2041% |
| Total Costs associated with Peritoneal Dialysis from the second year onwards | -67.8787% | -57.97% |
| Total Costs associated with Haemodialysis from the second year onwards | -67.8604% | -59.081% |
| Total Costs associated with Kidney Transplant from the second year onwards | -67.2557% | -58.6668% |
| Quality of life for Kidney Transplant patients with complications | -67.1117% | -60.6659% |
| Quality of life for Haemodialysis patients | -64.9811% | -58.6914% |
| Transition probability from Haemodialysis to Death | -66.9088% | -60.7874% |
| Total Costs associated with Peritoneal Dialysis in the first year | -66.0424% | -60.4102% |
| Transition probability from Peritoneal Dialysis to Death | -66.0478% | -60.7188% |

Figure 5: Tornado diagram for Scenario analysis between Scenario 1 and Scenario 2

Tornado diagram Societal perspective SCE 1- SCE 4

|  | Lower bound values | Upper bound values |
| --- | --- | --- |
| Discounting rate for outcomes | -39.5337% | 37.8912% |
| Discounting rate for costs | -25.4282% | 45.1757% |
| Total Costs associated with Peritoneal Dialysis from the second year onwards | -14.8981% | 11.3539% |
| Total Costs associated with Haemodialysis from the second year onwards | -14.8497% | 8.41047% |
| Total Costs associated with Kidney Transplant from the second year onwards | -13.2476% | 9.50789% |
| Quality of life for Kidney Transplant patients with complications | -10.5792% | 6.94659% |
| Transition probability from Haemodialysis to Death | -11.9398% | 3.55253% |
| Quality of life for Haemodialysis patients | -10.2798% | 4.74923% |
| Total Costs associated with Peritoneal Dialysis in the first year | -10.0329% | 4.88874% |
| Transition probability from Peritoneal Dialysis to Death | -9.86491% | 3.86107% |

Figure 6: Tornado diagram for Scenario analysis between Scenario 1 and Scenario 4

Figure 7: Tornado diagram for Scenario 3 compared to Scenario 1 during a 10- year time horizon

# D. **Results of probabilistic sensitivity analysis**

Cost-Effectiveness Plane Societal Perspective Pairwise analysis- lifetime horizon

Incremental Cost (KSHS)

| Incremental Effectiveness (QALY) | WTP Threshold |
| --- | --- |
| 0 | 0 |
| 20 | 2400000 |

| Incremental Effectiveness (QALY) | MEAN ICER |
| --- | --- |
| 7.76109 | 13916172 |
| 9.98323 | 20158501 |
| 21.245 | 19433303 |

| Incremental Effectiveness (QALY) | KT |
| --- | --- |
| 12.6103 | 3819847 |
| 12.331 | 4380906 |
| 14.9193 | 4021598 |
| 14.569 | 6448811 |
| 14.8696 | 6206030 |
| 13.9497 | 6637592 |
| 13.8 | 8240664 |
| 13.3219 | 3919976 |
| 13.565 | 6311200 |
| 14.4958 | 5289570 |
| 12.6604 | 6352149 |
| 15.2533 | 5338350 |
| 11.219 | 4796254 |
| 13.6855 | 5148638 |
| 13.6846 | 4676262 |
| 13.1022 | 4138196 |
| 14.1786 | 5828136 |
| 12.5173 | 3751779 |
| 15.4965 | 6319045 |
| 13.5051 | 6752471 |
| 12.5335 | 5754869 |
| 12.6144 | 6031342 |
| 12.4342 | 5782317 |
| 12.602 | 6339059 |
| 14.1496 | 5533482 |
| 11.5045 | 6067837 |
| 14.2507 | 5162973 |
| 14.2948 | 5776185 |
| 11.3877 | 8029612 |
| 12.7538 | 4314061 |
| 15.4994 | 6758550 |
| 15.026 | 4514852 |
| 15.6146 | 6515359 |
| 14.1908 | 6394298 |
| 12.9846 | 3936641 |
| 14.078 | 7004933 |
| 11.7107 | 3400308 |
| 11.2044 | 5086717 |
| 15.1045 | 7394056 |
| 14.5268 | 3870335 |
| 14.4143 | 4878722 |
| 13.5358 | 7388106 |
| 13.7479 | 4702032 |
| 14.4451 | 5552056 |
| 12.3269 | 4724077 |
| 12.8345 | 6482552 |
| 13.1459 | 4802398 |
| 13.9383 | 5800723 |
| 13.9587 | 5782584 |
| 13.6843 | 8069752 |
| 14.4443 | 5140572 |
| 12.2476 | 5354530 |
| 14.8028 | 6226746 |
| 14.3882 | 3942414 |
| 13.4664 | 4955008 |
| 11.7746 | 6899006 |
| 14.1891 | 3927898 |
| 14.3378 | 2634588 |
| 13.2061 | 8310192 |
| 13.5509 | 5764929 |
| 13.7048 | 3009571 |
| 12.7836 | 7241585 |
| 12.6146 | 3953266 |
| 14.7313 | 4276926 |
| 12.5476 | 6345714 |
| 14.5012 | 4603017 |
| 14.0143 | 4891996 |
| 12.1167 | 6934361 |
| 14.8608 | 8129764 |
| 12.8204 | 6799013 |
| 12.658 | 6363753 |
| 14.4037 | 6409336 |
| 13.0535 | 4643176 |
| 10.9605 | 6097206 |
| 13.8435 | 3707204 |
| 14.1283 | 4660733 |
| 13.7295 | 6192231 |
| 10.5383 | 3222109 |
| 13.0513 | 5824713 |
| 12.2853 | 2849278 |
| 13.3651 | 6402910 |
| 13.2157 | 5644243 |
| 12.2071 | 4466100 |
| 12.4597 | 6848711 |
| 12.7748 | 6389519 |
| 14.3145 | 6153144 |
| 11.907 | 6265230 |
| 13.4625 | 5093399 |
| 13.5133 | 4646207 |
| 13.9343 | 6673376 |
| 12.5341 | 6306762 |
| 14.2309 | 5313410 |
| 11.9427 | 7322852 |
| 11.6475 | 4305524 |
| 13.9182 | 4644896 |
| 12.3425 | 7706794 |
| 14.5691 | 5719349 |
| 12.6538 | 3996416 |
| 12.9285 | 6352622 |
| 14.0103 | 4824285 |
| 13.7688 | 4066235 |
| 14.8188 | 4830557 |
| 13.408 | 4203662 |
| 12.2736 | 7549142 |
| 13.9447 | 6052914 |
| 12.3812 | 4821273 |
| 12.2606 | 4528905 |
| 14.8307 | 6139946 |
| 13.6705 | 5984276 |
| 12.4975 | 6622538 |
| 12.3686 | 4125078 |
| 13.0368 | 2747276 |
| 12.2115 | 4688943 |
| 14.5069 | 7164081 |
| 12.1156 | 3479593 |
| 13.1415 | 5864132 |
| 15.2113 | 6493498 |
| 13.8898 | 4767967 |
| 14.2523 | 6089804 |
| 12.8614 | 8602245 |
| 13.9368 | 5396991 |
| 13.7386 | 5556707 |
| 12.3954 | 6864281 |
| 11.8746 | 5292523 |
| 12.6443 | 5191202 |
| 13.9605 | 5905778 |
| 12.9139 | 4875632 |
| 13.8962 | 6684099 |
| 14.7303 | 5972578 |
| 13.1214 | 3094151 |
| 13.5276 | 5013884 |
| 13.8924 | 4745848 |
| 13.0088 | 2558129 |
| 15.1708 | 6928977 |
| 13.4473 | 1692710 |
| 13.8482 | 3885302 |
| 12.5844 | 3974370 |
| 14.1358 | 6115051 |
| 13.5205 | 2938301 |
| 14.0827 | 7451213 |
| 13.4932 | 4097709 |
| 12.7578 | 5177719 |
| 12.8687 | 5569997 |
| 15.946 | 7971054 |
| 12.4246 | 3804890 |
| 14.0473 | 6036385 |
| 12.7369 | 5561813 |
| 14.4967 | 7561198 |
| 13.7991 | 4174805 |
| 15.1052 | 5706470 |
| 10.8786 | 5508059 |
| 15.3092 | 7296187 |
| 12.458 | 6472854 |
| 12.903 | 4467572 |
| 12.7437 | 3298606 |
| 12.1238 | 7076891 |
| 15.6777 | 4900522 |
| 12.666 | 3761138 |
| 13.7319 | 5803460 |
| 15.0171 | 4075610 |
| 12.6617 | 4937337 |
| 12.5963 | 5645275 |
| 11.8496 | 2345453 |
| 15.6268 | 6887377 |
| 13.4902 | 5429125 |
| 12.4735 | 4491892 |
| 14.486 | 6212470 |
| 12.576 | 5922219 |
| 14.2533 | 5934696 |
| 12.5296 | 6681297 |
| 13.9777 | 5551666 |
| 13.1837 | 5906659 |
| 15.3865 | 6725117 |
| 14.4771 | 5243975 |
| 14.759 | 5781437 |
| 13.9819 | 5567130 |
| 13.0922 | 6099695 |
| 14.8386 | 7160047 |
| 14.4479 | 3871641 |
| 13.3106 | 3950903 |
| 14.2214 | 5325306 |
| 13.0675 | 4774600 |
| 13.4438 | 4874375 |
| 14.6194 | 6624253 |
| 15.2923 | 5676829 |
| 15.4083 | 5367195 |
| 13.4778 | 4296309 |
| 15.1016 | 5826684 |
| 13.2137 | 4637344 |
| 13.7647 | 5916800 |
| 13.428 | 6529592 |
| 13.9836 | 5145276 |
| 13.8627 | 4246967 |
| 12.9937 | 4080978 |
| 13.7001 | 2183531 |
| 14.2781 | 4543244 |
| 12.4009 | 6761226 |
| 12.8022 | 4618277 |
| 14.9334 | 6715230 |
| 13.7898 | 7437275 |

| Incremental Effectiveness (QALY) | PD |
| --- | --- |
| 2.40242 | 6268816 |
| 2.24098 | 6966598 |
| 3.46526 | 4188541 |
| 2.98798 | 7118725 |
| 2.27547 | 5694388 |
| 3.73789 | 8965992 |
| 1.38 | 6323564 |
| 3.20737 | 5856304 |
| 3.25064 | 8203000 |
| 2.15536 | 6107792 |
| 3.02756 | 8870335 |
| 1.71186 | 5064911 |
| 1.12764 | 6303347 |
| 1.69448 | 4566413 |
| 2.38579 | 3232531 |
| 2.67724 | 7165194 |
| 3.04393 | 6502771 |
| 1.90874 | 7036905 |
| 3.75296 | 4160010 |
| 2.83263 | 7237597 |
| 2.21189 | 8755305 |
| 2.86452 | 5594268 |
| 1.69431 | 4104216 |
| 2.07432 | 8969473 |
| 1.57157 | 6474509 |
| 1.26874 | 6549160 |
| 2.70266 | 6346965 |
| 2.52026 | 5701669 |
| 0.667063 | 5950515 |
| 2.27694 | 6869945 |
| 2.49205 | 7269190 |
| 2.07294 | 5143075 |
| 3.50215 | 6591398 |
| 2.68314 | 6070929 |
| 2.24398 | 5454077 |
| 2.53271 | 6704822 |
| 1.97228 | 5074748 |
| 1.64075 | 5385039 |
| 1.02873 | 4394946 |
| 2.19878 | 7047225 |
| 2.36887 | 5716281 |
| 2.61053 | 9084639 |
| 2.55902 | 5676401 |
| 2.71748 | 5584421 |
| 0.444769 | 3474237 |
| 0.864855 | 5691829 |
| 2.43628 | 7089128 |
| 1.82575 | 7011908 |
| 1.85866 | 5798204 |
| 1.96704 | 9219286 |
| 2.17873 | 7427416 |
| 1.32702 | 3335964 |
| 2.7027 | 5332519 |
| 1.38989 | 4879037 |
| 2.08126 | 2766592 |
| 2.18068 | 4873581 |
| 2.35785 | 5025003 |
| 1.55218 | 4028177 |
| 2.97064 | 7822585 |
| 3.02186 | 6116910 |
| 2.45688 | 5679156 |
| 1.049 | 7813593 |
| 2.62238 | 6583146 |
| 2.03248 | 5393913 |
| 2.32979 | 8019317 |
| 2.76641 | 8702894 |
| 2.94676 | 6527515 |
| 1.37948 | 6535288 |
| 2.91821 | 9469877 |
| 1.98341 | 7743956 |
| 2.21385 | 5497065 |
| 2.55502 | 10647594 |
| 2.0409 | 5833454 |
| 3.36485 | 9114177 |
| 2.21247 | 5323659 |
| 1.80925 | 5319092 |
| 1.83853 | 5243143 |
| 1.31361 | 6399923 |
| 2.35767 | 4568732 |
| 1.33267 | 3344332 |
| 2.73576 | 5564084 |
| 2.42903 | 5122006 |
| 2.30586 | 5131893 |
| 2.85458 | 10386246 |
| 1.50311 | 5506887 |
| 2.7957 | 6296903 |
| 0.0694771 | 4840517 |
| 2.73697 | 5924500 |
| 1.54114 | 3309204 |
| 2.55721 | 6759576 |
| 2.88435 | 7364809 |
| 2.63011 | 5973106 |
| 2.82067 | 7050789 |
| 1.54828 | 4900690 |
| 3.42039 | 7867541 |
| 2.20843 | 8662752 |
| 1.78655 | 4373069 |
| 2.41692 | 8376674 |
| 1.45458 | 6821166 |
| 1.25895 | 6846447 |
| 1.76868 | 6948069 |
| 2.3242 | 6821410 |
| 0.717542 | 1915962 |
| 1.4231 | 3415778 |
| 2.627 | 8139708 |
| 2.70784 | 10321417 |
| 2.14792 | 5186685 |
| 1.57988 | 5200282 |
| 3.31707 | 7419469 |
| 2.03406 | 6087856 |
| 0.470797 | 3816872 |
| 0.782865 | 2887933 |
| 2.11991 | 6168752 |
| 3.10712 | 6268637 |
| 1.99228 | 5362706 |
| 1.994 | 6334515 |
| 2.23106 | 7488147 |
| 2.61943 | 6124784 |
| 1.95871 | 5609776 |
| 2.38529 | 11004781 |
| 2.03005 | 4818414 |
| 1.80899 | 7145185 |
| 2.08603 | 7947728 |
| 1.82181 | 6647762 |
| 1.51045 | 6348520 |
| 2.86005 | 7584069 |
| 1.91492 | 5847248 |
| 2.68997 | 5850103 |
| 1.74171 | 5408573 |
| 1.88025 | 4720880 |
| 3.03195 | 8166006 |
| 1.06319 | 6100522 |
| 1.69778 | 4807127 |
| 1.90109 | 5362462 |
| 2.23186 | 5305988 |
| 2.49725 | 6179611 |
| 1.51794 | 5562503 |
| 2.18 | 9187123 |
| 1.34091 | 3191470 |
| 3.18067 | 8307781 |
| 3.26672 | 8397879 |
| 1.91437 | 6643606 |
| 1.07214 | 7566909 |
| 4.31255 | 12217890 |
| 2.36344 | 6956509 |
| 3.05914 | 9960745 |
| 2.30324 | 4854548 |
| 1.92762 | 6217276 |
| 1.61628 | 4020549 |
| 2.31676 | 7149504 |
| 2.10726 | 8442877 |
| 3.00462 | 8268421 |
| 2.90702 | 8423706 |
| 2.33736 | 7022191 |
| 1.05064 | 3771749 |
| 2.1129 | 7171803 |
| 2.89498 | 8604089 |
| 0.948917 | 2403966 |
| 1.99717 | 8570402 |
| 2.24176 | 6072606 |
| 1.89237 | 5088024 |
| 2.25154 | 9683023 |
| 1.83028 | 7403463 |
| 3.05578 | 7367679 |
| 2.47634 | 5465676 |
| 1.29274 | 4326252 |
| 3.83588 | 7772203 |
| 0.952192 | 5657801 |
| 2.60302 | 6210170 |
| 2.39167 | 7722048 |
| 2.60312 | 4879376 |
| 2.08914 | 6563466 |
| 3.01296 | 7348915 |
| 1.35727 | 4977546 |
| 1.7233 | 3058023 |
| 2.4252 | 4537128 |
| 2.54599 | 6938245 |
| 3.32069 | 6681326 |
| 3.90304 | 5343538 |
| 1.94507 | 7160886 |
| 2.62371 | 5989471 |
| 2.64003 | 6106977 |
| 1.76908 | 6398580 |
| 2.3344 | 4954812 |
| 2.28577 | 6756695 |
| 3.73387 | 6802079 |
| 2.85247 | 6639399 |
| 3.51707 | 6523624 |
| 0.998011 | 6032614 |
| 3.10147 | 7350284 |
| 1.37775 | 8496676 |
| 3.24005 | 7207323 |
| 1.50995 | 6311909 |
| 2.3223 | 5320710 |
| 2.04225 | 4763984 |
| 2.04257 | 5556797 |
| 2.89648 | 6558652 |
| 1.45144 | 6516530 |
| 2.77049 | 8028225 |
| 1.26579 | 7512847 |

| Incremental Effectiveness (QALY) | HD |
| --- | --- |
| -0.703289 | 483171 |
| -0.980632 | -1094879 |
| -0.571227 | -2209555 |
| 0.736794 | 398050 |
| -0.495466 | -1681530 |
| 1.6321 | 2222406 |
| -0.476106 | 270078 |
| 0.395055 | -701765 |
| 0.649356 | -198334 |
| -0.0803448 | -530202 |
| 0.202169 | 1394841 |
| -0.35764 | -1209977 |
| -1.07967 | -991752 |
| 0.109436 | -765815 |
| 0.597969 | 600667 |
| 0.0338383 | -53125.6 |
| 0.36498 | -219925 |
| -0.5254 | -90576.8 |
| 0.29224 | -2103899 |
| 1.18286 | 856662 |
| -1.61874 | -1352043 |
| 1.50331 | 1630695 |
| 0.979874 | -799917 |
| 0.493435 | 2115677 |
| -0.817163 | -1747758 |
| -0.25945 | -64056.4 |
| 0.798134 | 643940 |
| 0.445044 | 1898846 |
| -0.666187 | 135485 |
| -0.275997 | 1015763 |
| 0.575852 | 576826 |
| -0.118001 | -217003 |
| 1.38035 | 478237 |
| -0.77564 | -257121 |
| -0.514017 | -307902 |
| -0.0653251 | 1713781 |
| -0.418515 | -1150286 |
| -0.268989 | -1194243 |
| 0.391751 | 1350032 |
| -0.209886 | -7306.56 |
| 0.133321 | 108680 |
| 0.591003 | 1112779 |
| -0.116046 | -652537 |
| 0.713056 | -128602 |
| -0.892148 | -1641082 |
| -0.564028 | -921636 |
| -0.558921 | -990043 |
| -0.382626 | -212776 |
| 0.182014 | -174108 |
| -0.84846 | 2356144 |
| -0.635617 | 738884 |
| -1.02937 | -1419346 |
| 0.5362 | 1009963 |
| -0.847492 | -2229692 |
| 0.346488 | -2763007 |
| 0.529768 | 203881 |
| 0.123763 | -1157285 |
| -0.788617 | -1881103 |
| 1.59605 | 1691957 |
| 0.724854 | 774451 |
| -0.154223 | -66847.1 |
| -0.500549 | 519053 |
| 0.118383 | 251460 |
| -0.326047 | -1943729 |
| 0.652467 | 2429609 |
| 0.771297 | 2101204 |
| 0.844534 | 725276 |
| -0.831594 | 277478 |
| 0.362292 | 1472968 |
| 0.64267 | 2207871 |
| 0.257446 | 560778 |
| 0.0971171 | 1165405 |
| -0.728502 | -578062 |
| -0.125157 | 407683 |
| 0.174483 | -1177368 |
| -0.23913 | 883271 |
| 0.382992 | -337583 |
| -0.971764 | -1654921 |
| -0.0346286 | -810563 |
| -1.31509 | -2272458 |
| -0.172075 | -462595 |
| 0.60954 | 96800.3 |
| -0.24381 | -1318904 |
| -0.476572 | 1208273 |
| -0.675423 | 191286 |
| -0.12422 | 965600 |
| -0.995768 | 1566208 |
| 0.723089 | -282095 |
| -0.0638936 | -2851382 |
| 0.367343 | 594477 |
| -0.1457 | 359385 |
| -0.154354 | -1431156 |
| -0.439948 | 585267 |
| -0.984137 | -1515677 |
| 0.190081 | -1310136 |
| -0.51483 | 1987540 |
| 0.528486 | -382162 |
| 0.143147 | -55373.2 |
| -1.39866 | -165961 |
| -0.0330728 | 283109 |
| -0.315137 | -160943 |
| 1.19171 | 77061 |
| -1.084 | -3045225 |
| -0.593537 | 450239 |
| 0.56168 | 1395201 |
| 0.275898 | 1658410 |
| -0.967408 | -2816016 |
| 0.0257201 | 501363 |
| 0.807851 | 733158 |
| -0.227056 | -465249 |
| -1.2387 | -2009304 |
| -0.880647 | -963582 |
| -1.29754 | -1282296 |
| 0.301566 | -667279 |
| -0.107466 | -1605689 |
| 0.313695 | 510735 |
| 0.414731 | 885832 |
| -0.66844 | -2544177 |
| 0.734506 | 373494 |
| 0.255015 | 4026189 |
| 1.17539 | 279861 |
| -0.00804644 | 169136 |
| 1.07831 | 2061576 |
| -0.450449 | 896013 |
| 0.590773 | 2024289 |
| 1.07882 | 2516862 |
| -0.208177 | -476240 |
| 1.86987 | 1763428 |
| 0.194333 | 252558 |
| -0.61798 | -1492133 |
| -0.421759 | -1075820 |
| -0.0419704 | 826150 |
| -0.0486839 | -56064.8 |
| -0.0972634 | 1311387 |
| -1.06347 | -1920400 |
| 0.0711854 | -430565 |
| -0.328474 | -683904 |
| -0.231414 | 509469 |
| -1.13498 | -2096238 |
| 0.927658 | 2147736 |
| 0.681248 | 1686684 |
| 0.0216947 | 226527 |
| -1.00683 | 213826 |
| 1.14875 | 1284737 |
| 1.01321 | 1076928 |
| 0.669315 | 2415645 |
| 0.684302 | -771704 |
| -0.281266 | -569248 |
| -0.115465 | -448603 |
| 0.9473 | 1001573 |
| -0.629266 | -386666 |
| 0.544573 | 751532 |
| -0.317586 | 566846 |
| 0.728165 | 1044574 |
| -0.657082 | -1447535 |
| 1.34037 | 2545391 |
| 0.279239 | -214789 |
| -0.581365 | -1624869 |
| -1.04285 | -853492 |
| 0.778708 | -307030 |
| 0.125087 | -118094 |
| -0.160792 | 2868521 |
| -1.10778 | -2175761 |
| 0.767154 | 794685 |
| 0.549604 | 115451 |
| -1.37752 | -1246096 |
| 0.29101 | 605330 |
| -0.189671 | -684992 |
| 1.26624 | 690050 |
| -0.632815 | -1537708 |
| -1.0632 | -1572988 |
| -0.0193456 | 694737 |
| 0.902366 | 2372513 |
| 0.136719 | -478502 |
| -0.903492 | -2654031 |
| 0.263055 | -1118247 |
| 0.78704 | 717095 |
| 1.5377 | 1067909 |
| -0.566311 | -1208928 |
| -0.394361 | 373542 |
| 1.16533 | 1286129 |
| 0.372797 | 1706796 |
| -0.187798 | -882742 |
| -0.762354 | -1200291 |
| -0.907574 | -169688 |
| 0.154496 | 1084420 |
| 0.00439669 | 112873 |
| 1.19116 | 1074158 |
| -0.0684979 | 1606359 |
| -0.137729 | -1941451 |
| -0.0347158 | 2330834 |
| 0.741321 | 70814.9 |
| 0.945888 | -55973.6 |
| -0.520009 | -1111789 |
| -0.138641 | -1564638 |
| 0.419014 | 164379 |
| 0.350254 | -391986 |
| -1.25733 | -623791 |
| 0.730883 | 1948385 |
| 0.000306083 | 578007 |

Figure 8: Cost-effectiveness plane- Pairwise approach (Societal perspective)

Cost-Effectiveness Plane Societal Perspective Scenario approach- lifetime horizon

Incremental Cost (KSHS)

| Incremental Effectiveness (QALY) | SCE 1 |
| --- | --- |
| -1.65986 | 116060 |
| -1.00024 | -1221378 |
| 0.0901077 | 194014 |
| 0.725137 | 760544 |
| 0.745855 | -652905 |
| -0.797089 | -509854 |
| 0.871741 | -255666 |
| 0.00695495 | -728203 |
| 0.268002 | -790542 |
| 0.379929 | -888893 |
| 0.756175 | 2311423 |
| -0.362068 | 1034272 |
| 0.103596 | 1300746 |
| -0.467002 | -26081 |
| -0.275871 | -206752 |
| 0.110791 | 602492 |
| 0.726618 | 1135663 |
| 0.835428 | 1940661 |
| -0.338672 | -145233 |
| 0.141727 | -807580 |
| -1.04074 | 548024 |
| 0.403453 | 2307846 |
| -0.055748 | 1762942 |
| -0.666953 | -1758916 |
| 0.0961973 | 553433 |
| 0.176548 | -475483 |
| 0.334126 | 1198817 |
| 0.198392 | -616088 |
| 0.531383 | 2033828 |
| 1.29772 | 853625 |
| -0.90305 | -2071966 |
| 1.24139 | 294852 |
| 0.283049 | 1311166 |
| -0.023525 | 630119 |
| -0.106422 | 338697 |
| -1.4353 | -3756165 |
| 0.968926 | 106482 |
| -0.545825 | -232019 |
| 0.0839454 | -775776 |
| -1.0051 | -1875965 |
| 0.834087 | -1427694 |
| 0.0720105 | 1158041 |
| -0.312976 | -518895 |
| -0.657949 | -1457463 |
| 1.09432 | -180536 |
| -0.214353 | 432762 |
| 0.305051 | 833485 |
| 0.111769 | 1671792 |
| 0.0118402 | -375074 |
| 0.269103 | -732980 |
| 0.376633 | -941155 |
| -0.59282 | -778009 |
| 0.168011 | -153312 |
| -0.755218 | -341745 |
| -0.915736 | -910045 |
| 0.808267 | 1865154 |
| -0.500097 | -33041.2 |
| -0.117609 | 807655 |
| 0.106017 | -339701 |
| -0.392691 | 621142 |
| -0.229419 | -1167041 |
| 0.30936 | 681136 |
| -0.176285 | -101100 |
| -0.627034 | -737420 |
| 0.065848 | 3308507 |
| -0.010872 | 785396 |
| -0.555642 | -392824 |
| -0.367148 | -1035521 |
| 1.1774 | 995062 |
| 0.421428 | -531762 |
| -0.662804 | -554737 |
| 0.195012 | -436465 |
| 0.0625106 | -292558 |
| 0.608124 | 815710 |
| 0.248729 | 389793 |
| 0.0520759 | -416562 |
| 0.604569 | 830937 |
| -1.19687 | -1755713 |
| -1.55529 | -367287 |
| 0.996035 | 333142 |
| -0.446206 | -724885 |
| -0.282362 | -215978 |
| 0.578265 | 1974699 |
| -0.44774 | -2275798 |
| -0.529223 | -47815.8 |
| -0.907528 | -789977 |
| -0.843848 | -1886025 |
| -0.339289 | -932693 |
| 1.02797 | 846709 |
| 0.121204 | 71233.9 |
| -0.192524 | 1268889 |
| -0.0284925 | -443883 |
| -0.884107 | -2357021 |
| -1.01622 | -975931 |
| 1.27818 | 1550786 |
| -1.33125 | -1114657 |
| 1.11403 | -617970 |
| -0.11079 | -909053 |
| -0.186648 | -1119386 |
| -1.21826 | -335324 |
| -0.748857 | -2044868 |
| -0.0479211 | 296937 |
| 0.177602 | -159660 |
| 0.450921 | 954409 |
| 0.127153 | 837544 |
| -0.477089 | -959073 |
| -0.215469 | -927006 |
| 0.0251685 | 374568 |
| -1.01394 | -394292 |
| -0.113171 | -11213 |
| 0.47225 | 242286 |
| -0.142529 | 1315842 |
| -0.461147 | -731524 |
| 0.506196 | 619728 |
| -0.0475554 | -830313 |
| 0.50187 | 875309 |
| -0.809693 | -2051368 |
| 0.608515 | 529703 |
| 0.384351 | 995744 |
| 0.125399 | 1766111 |
| 0.365976 | 748440 |
| 0.798861 | 4230288 |
| 0.428033 | -1218825 |
| 0.145332 | 1325535 |
| -1.2945 | -1008857 |
| 0.552337 | 1230962 |
| -1.45093 | -3007412 |
| -0.365549 | 275256 |
| 0.261351 | -196223 |
| -1.14286 | -2146243 |
| -0.39511 | -347893 |
| 0.362751 | -757468 |
| 0.131384 | 40823.7 |
| 1.19185 | 573614 |
| -1.37472 | -3010974 |
| -0.0112902 | 553837 |
| -0.479869 | -809.029 |
| -0.617598 | 111278 |
| 1.4226 | -279643 |
| -0.545464 | 82382.1 |
| -0.549333 | -1947473 |
| -0.437081 | 438652 |
| -0.313444 | 73506.9 |
| 0.846564 | 1790340 |
| -0.391875 | -592525 |
| -0.0741092 | 253066 |
| -0.546505 | -1146087 |
| 0.416321 | 632406 |
| -0.261499 | -1057486 |
| 0.59269 | -558415 |
| -0.22308 | 14756 |
| -0.346499 | 1646663 |
| -1.16534 | -1777888 |
| 0.59122 | 1681575 |
| 0.124497 | 1347122 |
| 0.539415 | -662045 |
| -0.288518 | 199619 |
| 0.077705 | 894558 |
| -0.88006 | -613262 |
| 0.154615 | 170071 |
| 0.0643532 | 1477656 |
| 0.27199 | 1120528 |
| -0.484574 | -1721807 |
| 0.80357 | 975320 |
| -0.383876 | -1320502 |
| -0.0983553 | 271480 |
| -0.151219 | -264874 |
| 0.29526 | -433111 |
| 0.908843 | -1141892 |
| 0.0714271 | 834771 |
| -0.437174 | -256513 |
| -0.515042 | -1229286 |
| 1.23309 | 66346.8 |
| -0.0640025 | 995121 |
| 0.240818 | 1967886 |
| -0.923729 | -601426 |
| -1.46164 | -1316210 |
| 0.438476 | -296521 |
| -0.996047 | -1393394 |
| -0.25383 | 1603966 |
| 0.470176 | 1132659 |
| -0.883146 | -1346789 |
| -0.00456534 | 651030 |
| 0.824387 | 485668 |
| 1.48485 | 1210146 |
| 0.027686 | 1363566 |
| 0.432211 | -1176402 |
| 0.180904 | 1958667 |
| -0.584394 | -1167620 |
| -0.69935 | -1579142 |
| 0.231778 | 64289.6 |
| 1.98234 | 1879307 |
| -0.725772 | 167379 |
| -1.1994 | -734117 |
| -1.28287 | -1343819 |
| -0.588801 | -121878 |
| -0.924704 | -1330304 |
| 1.36702 | 680805 |
| 0.00696625 | -546204 |
| 1.37288 | 2663327 |

| Incremental Effectiveness (QALY) | SCE 2 |
| --- | --- |
| -1.39358 | 708165 |
| -0.848579 | -755102 |
| 0.362147 | 825398 |
| 0.759573 | 1210731 |
| 0.972987 | -131932 |
| -0.563239 | 119892 |
| 0.957467 | 162741 |
| 0.182937 | -179597 |
| 0.423559 | -121995 |
| 0.520861 | -483375 |
| 0.931927 | 2785681 |
| -0.134057 | 1611096 |
| 0.296473 | 1903001 |
| -0.297905 | 695882 |
| -0.159067 | 196288 |
| 0.263873 | 1176909 |
| 0.988915 | 1722524 |
| 1.07311 | 2538570 |
| -0.178283 | 423716 |
| 0.386993 | 12519.1 |
| -0.706332 | 983897 |
| 0.565369 | 2751085 |
| 0.0715012 | 2137316 |
| -0.452314 | -1262999 |
| 0.275737 | 1103788 |
| 0.289132 | -139527 |
| 0.535208 | 1720500 |
| 0.353156 | -68677.4 |
| 0.661681 | 2563721 |
| 1.39851 | 1336553 |
| -0.658187 | -1517808 |
| 1.39373 | 722027 |
| 0.5786 | 1994456 |
| 0.208912 | 1349759 |
| 0.142679 | 867373 |
| -1.09811 | -2989237 |
| 1.14262 | 608377 |
| -0.37092 | 165746 |
| 0.235915 | -229151 |
| -0.855014 | -1415615 |
| 0.989446 | -870829 |
| 0.244034 | 1593168 |
| -0.137052 | -84742.3 |
| -0.458441 | -1087020 |
| 1.24238 | 356341 |
| -0.0549018 | 864804 |
| 0.368825 | 1167228 |
| 0.264463 | 2268008 |
| 0.261011 | 294747 |
| 0.480937 | -47461.8 |
| 0.570083 | -469942 |
| -0.381617 | -261952 |
| 0.43587 | 356006 |
| -0.48 | 531523 |
| -0.701992 | -216959 |
| 0.955596 | 2224426 |
| -0.247958 | 523875 |
| 0.127004 | 1505656 |
| 0.31327 | 99113.9 |
| -0.232514 | 1165023 |
| -0.0752861 | -677734 |
| 0.511851 | 1241189 |
| 0.0704604 | 556179 |
| -0.335001 | -58610.4 |
| 0.299115 | 3766959 |
| 0.214838 | 1394626 |
| -0.343604 | 150937 |
| -0.037221 | -274683 |
| 1.31418 | 1485644 |
| 0.554448 | 90367 |
| -0.364402 | 44293.8 |
| 0.386287 | 104795 |
| 0.303185 | 293383 |
| 0.755676 | 1266463 |
| 0.531105 | 995255 |
| 0.12751 | -66624.4 |
| 0.785678 | 1522268 |
| -0.959775 | -1202358 |
| -1.19625 | 324064 |
| 1.12336 | 778418 |
| -0.285512 | -253008 |
| 0.0169784 | 591862 |
| 0.707826 | 2572270 |
| -0.230532 | -1843399 |
| -0.326911 | 465178 |
| -0.707964 | -300158 |
| -0.586119 | -1133774 |
| -0.119592 | -404745 |
| 1.18057 | 1449743 |
| 0.298172 | 692454 |
| -0.0551719 | 1712460 |
| 0.0661186 | -114836 |
| -0.722135 | -1757845 |
| -0.806389 | -262463 |
| 1.46743 | 2040143 |
| -1.05833 | -455695 |
| 1.26941 | -85995.1 |
| 0.0386526 | -426653 |
| -0.128311 | -794523 |
| -1.05249 | 89648.6 |
| -0.516707 | -1358693 |
| 0.12623 | 957840 |
| 0.497211 | 429547 |
| 0.607111 | 1515010 |
| 0.248413 | 1362879 |
| -0.223854 | -525210 |
| -0.081183 | -263751 |
| 0.293522 | 924743 |
| -0.741937 | 280497 |
| 0.042412 | 482092 |
| 0.669705 | 770131 |
| 0.0430459 | 1836110 |
| -0.273411 | -208087 |
| 0.708759 | 1261296 |
| 0.176188 | -124794 |
| 0.682605 | 1578784 |
| -0.55923 | -1295909 |
| 0.758622 | 1003269 |
| 0.52804 | 1518352 |
| 0.367637 | 2461099 |
| 0.613304 | 1237783 |
| 0.970625 | 4835396 |
| 0.6338 | -792791 |
| 0.356931 | 1898670 |
| -1.05627 | -466192 |
| 0.72022 | 1764063 |
| -1.19429 | -2416324 |
| -0.0551263 | 954129 |
| 0.434871 | 335718 |
| -0.80441 | -1421715 |
| -0.207777 | 318485 |
| 0.516133 | -362744 |
| 0.266256 | 624329 |
| 1.25299 | 1121649 |
| -1.18885 | -2385599 |
| 0.161604 | 1007560 |
| -0.294532 | 547334 |
| -0.414682 | 617672 |
| 1.50645 | 294494 |
| -0.364003 | 544360 |
| -0.281079 | -1208160 |
| -0.176718 | 1196302 |
| -0.0917096 | 509611 |
| 0.998537 | 2227523 |
| -0.156117 | 71768 |
| 0.132588 | 922818 |
| -0.305088 | -637238 |
| 0.601385 | 1032618 |
| -0.115252 | -605148 |
| 0.751387 | 85123.4 |
| -0.0368036 | 711152 |
| -0.249806 | 1915988 |
| -0.918099 | -1132825 |
| 0.755027 | 2023779 |
| 0.339581 | 2221999 |
| 0.697878 | 13633 |
| -0.0796119 | 452741 |
| 0.329591 | 1649235 |
| -0.570537 | 58247.7 |
| 0.334818 | 706060 |
| 0.193439 | 2013623 |
| 0.458691 | 1796189 |
| -0.197098 | -1166512 |
| 1.08133 | 1733193 |
| -0.0910779 | -818512 |
| 0.0339481 | 815913 |
| 0.13971 | 405933 |
| 0.49694 | 46101.2 |
| 1.05525 | -533711 |
| 0.281737 | 1395569 |
| -0.267215 | 168984 |
| -0.303128 | -541614 |
| 1.36811 | 506000 |
| 0.178299 | 1396620 |
| 0.442853 | 2385054 |
| -0.723543 | 6678.08 |
| -1.18559 | -683298 |
| 0.633983 | 254035 |
| -0.742496 | -849572 |
| -0.0561354 | 2243105 |
| 0.646178 | 1674785 |
| -0.669231 | -774667 |
| 0.201513 | 1149122 |
| 1.07516 | 1068053 |
| 1.67733 | 1720261 |
| 0.31656 | 2006060 |
| 0.622802 | -766583 |
| 0.361049 | 2445824 |
| -0.307833 | -465383 |
| -0.492144 | -988783 |
| 0.305149 | 362389 |
| 2.07054 | 2170184 |
| -0.465169 | 754200 |
| -0.861506 | 10579.1 |
| -1.05011 | -663232 |
| -0.341405 | 541516 |
| -0.686802 | -735883 |
| 1.56008 | 1296417 |
| 0.161481 | -47671.5 |
| 1.50516 | 3104888 |

| Incremental Effectiveness (QALY) | SCE 3 |
| --- | --- |
| -0.382552 | 592661 |
| 0.175062 | -889460 |
| 1.25778 | 560850 |
| 1.82238 | 1080996 |
| 1.9725 | -286108 |
| 0.503515 | -18300 |
| 1.91353 | 165308 |
| 1.17026 | -274923 |
| 1.51767 | -352050 |
| 1.48703 | -425038 |
| 1.94835 | 2576459 |
| 0.825468 | 1254464 |
| 1.35263 | 1606994 |
| 0.864321 | 417073 |
| 0.894081 | 143709 |
| 1.23425 | 986748 |
| 2.0797 | 1368881 |
| 2.18278 | 2066760 |
| 0.767973 | 226214 |
| 1.38093 | -226723 |
| 0.224888 | 766643 |
| 1.71233 | 2639475 |
| 1.15008 | 2005626 |
| 0.514019 | -1406745 |
| 1.15767 | 800986 |
| 1.48636 | -179964 |
| 1.53338 | 1466166 |
| 1.23705 | -241683 |
| 1.71842 | 2268623 |
| 2.35667 | 1081447 |
| 0.356051 | -1655198 |
| 2.4743 | 568825 |
| 1.49075 | 1648799 |
| 1.06619 | 1119152 |
| 1.26505 | 684152 |
| -0.0432958 | -3124292 |
| 2.14221 | 623428 |
| 0.620123 | 184752 |
| 1.33055 | -400617 |
| 0.346067 | -1339966 |
| 2.10854 | -1086345 |
| 1.25334 | 1334823 |
| 0.924546 | -24821.2 |
| 0.666722 | -922402 |
| 2.24728 | 134306 |
| 0.997462 | 802679 |
| 1.48184 | 893541 |
| 1.35931 | 1914560 |
| 1.23096 | -3306.99 |
| 1.51504 | -320919 |
| 1.64029 | -621587 |
| 0.525918 | -427794 |
| 1.54532 | 177020 |
| 0.389154 | 149009 |
| 0.273635 | -420432 |
| 2.08846 | 2210649 |
| 0.696466 | 380194 |
| 1.13227 | 1134616 |
| 1.15821 | -13645.7 |
| 0.961825 | 1004523 |
| 0.966547 | -702259 |
| 1.63286 | 987127 |
| 1.15114 | 280504 |
| 0.593754 | -291418 |
| 1.28583 | 3519142 |
| 0.893634 | 1057088 |
| 0.739714 | 41711.5 |
| 0.829904 | -614108 |
| 2.32898 | 1322703 |
| 1.6975 | -123074 |
| 0.760282 | -20385.8 |
| 1.41098 | -140761 |
| 1.31645 | -60978.8 |
| 1.77748 | 903106 |
| 1.54334 | 840984 |
| 1.29582 | -26811.3 |
| 1.95412 | 1192967 |
| 0.234142 | -1180392 |
| -0.194758 | -8416.85 |
| 2.05309 | 700863 |
| 0.866804 | -385654 |
| 0.913958 | 188063 |
| 1.7596 | 2145102 |
| 0.812688 | -1810945 |
| 0.709715 | 371649 |
| 0.377235 | -470135 |
| 0.497696 | -1468661 |
| 0.881436 | -735707 |
| 2.21726 | 1164605 |
| 1.26701 | 428899 |
| 0.930538 | 1450436 |
| 1.22985 | 63188.4 |
| 0.478821 | -1787421 |
| 0.154933 | -602935 |
| 2.38945 | 1781248 |
| -0.0715662 | -634450 |
| 2.51732 | -157169 |
| 1.11554 | -551245 |
| 0.944748 | -720206 |
| 0.0129453 | -84833.2 |
| 0.487532 | -1486884 |
| 1.16762 | 677855 |
| 1.41121 | 72784.2 |
| 1.64826 | 1213641 |
| 1.41004 | 1230740 |
| 0.862517 | -538059 |
| 0.854368 | -464911 |
| 1.36267 | 618962 |
| 0.388225 | 42408.6 |
| 1.1659 | 499655 |
| 1.80091 | 720658 |
| 1.20345 | 1520939 |
| 0.669385 | -404964 |
| 1.75435 | 896232 |
| 1.05027 | -405582 |
| 1.76608 | 1271697 |
| 0.531772 | -1561495 |
| 1.69151 | 865837 |
| 1.35404 | 1261788 |
| 1.31498 | 2128244 |
| 1.49593 | 887901 |
| 2.03925 | 4467533 |
| 1.59564 | -836123 |
| 1.36791 | 1687963 |
| -0.0678125 | -474596 |
| 1.701 | 1550815 |
| -0.258373 | -2464579 |
| 1.03107 | 874231 |
| 1.55239 | 229128 |
| 0.281439 | -1663590 |
| 0.882963 | 223703 |
| 1.55612 | -445117 |
| 1.33785 | 495770 |
| 2.29434 | 903215 |
| -0.0774298 | -2484929 |
| 1.2498 | 981383 |
| 0.761859 | 367872 |
| 0.684485 | 504755 |
| 2.47446 | 180486 |
| 0.56994 | 436312 |
| 0.78173 | -1510115 |
| 0.748359 | 803730 |
| 1.02802 | 451199 |
| 1.88435 | 2013629 |
| 0.841329 | -151347 |
| 1.07921 | 599889 |
| 0.619244 | -646299 |
| 1.76227 | 1108197 |
| 1.02602 | -561263 |
| 1.74891 | -164771 |
| 1.03697 | 357128 |
| 0.717489 | 1898094 |
| 0.107343 | -1415174 |
| 1.84197 | 1982914 |
| 1.33532 | 1693302 |
| 1.80521 | -201397 |
| 1.27444 | 495861 |
| 1.58073 | 1409626 |
| 0.274819 | -153384 |
| 1.34204 | 797759 |
| 1.14355 | 1899492 |
| 1.46233 | 1453002 |
| 0.710929 | -1340959 |
| 2.05083 | 1312031 |
| 0.988035 | -764556 |
| 1.05177 | 670638 |
| 1.31926 | 205627 |
| 1.48329 | -17361.6 |
| 2.2005 | -722924 |
| 1.17731 | 1153927 |
| 0.728499 | 219460 |
| 0.789123 | -840893 |
| 2.28916 | 434938 |
| 1.2069 | 1364204 |
| 1.54838 | 2134409 |
| 0.360703 | -145884 |
| -0.174636 | -785053 |
| 1.63287 | 335992 |
| 0.418506 | -804691 |
| 0.958495 | 1820193 |
| 1.97918 | 1519149 |
| 0.174456 | -973460 |
| 1.34611 | 1173668 |
| 1.9067 | 812376 |
| 2.71432 | 1382723 |
| 1.41246 | 1755332 |
| 1.62779 | -728787 |
| 1.43431 | 2480629 |
| 0.73381 | -694456 |
| 0.447815 | -1176771 |
| 1.42163 | 452880 |
| 3.14584 | 2012466 |
| 0.705157 | 474867 |
| 0.0691212 | -424687 |
| 0.151033 | -897119 |
| 0.620314 | 213373 |
| 0.329065 | -832013 |
| 2.62232 | 1041948 |
| 1.33239 | -162832 |
| 2.57105 | 2802807 |

| Incremental Effectiveness (QALY) | SCE 4 |
| --- | --- |
| 0.175958 | 1969395 |
| 0.487261 | 327688 |
| 1.83017 | 2021169 |
| 1.88422 | 2171640 |
| 2.45055 | 956297 |
| 0.993423 | 1490669 |
| 2.08543 | 1181478 |
| 1.53615 | 1004767 |
| 1.83961 | 1215481 |
| 1.77902 | 597920 |
| 2.31359 | 3764882 |
| 1.30467 | 2660500 |
| 1.75544 | 3047402 |
| 1.21281 | 2065876 |
| 1.13226 | 1107686 |
| 1.55087 | 2342783 |
| 2.6309 | 2780524 |
| 2.6831 | 3548811 |
| 1.10015 | 1606413 |
| 1.89749 | 1651405 |
| 0.932655 | 1838247 |
| 2.04676 | 3774612 |
| 1.41081 | 2959940 |
| 0.963583 | -198782 |
| 1.53277 | 2095612 |
| 1.71529 | 677063 |
| 1.9537 | 2735205 |
| 1.55781 | 1069567 |
| 1.98608 | 3553812 |
| 2.5623 | 2208066 |
| 0.868638 | -360320 |
| 2.79104 | 1644462 |
| 2.11477 | 3263297 |
| 1.55433 | 2816717 |
| 1.78616 | 1982288 |
| 0.667691 | -1380193 |
| 2.50387 | 1791753 |
| 0.984915 | 1145724 |
| 1.64353 | 881304 |
| 0.654215 | -281405 |
| 2.43002 | 249595 |
| 1.61048 | 2453953 |
| 1.28968 | 1059725 |
| 1.08275 | 44631.6 |
| 2.55375 | 1464543 |
| 1.32691 | 1901332 |
| 1.60561 | 1775730 |
| 1.67339 | 3376835 |
| 1.75447 | 1550051 |
| 1.95795 | 1278075 |
| 2.04374 | 595624 |
| 0.967409 | 776165 |
| 2.10846 | 1464520 |
| 0.970396 | 2169029 |
| 0.720466 | 1191129 |
| 2.39261 | 3062984 |
| 1.22696 | 1746771 |
| 1.64524 | 2825334 |
| 1.59227 | 1023455 |
| 1.29138 | 2303910 |
| 1.28582 | 471728 |
| 2.05425 | 2344219 |
| 1.66886 | 1844882 |
| 1.20949 | 1266613 |
| 1.77531 | 4656613 |
| 1.37077 | 2511201 |
| 1.18263 | 1369363 |
| 1.52711 | 1133210 |
| 2.61297 | 2520733 |
| 1.96998 | 1350317 |
| 1.38844 | 1421373 |
| 1.80922 | 1177509 |
| 1.82155 | 1358364 |
| 2.083 | 2041155 |
| 2.13814 | 2237104 |
| 1.44458 | 879427 |
| 2.33195 | 2832940 |
| 0.729671 | 106319 |
| 0.564139 | 1651641 |
| 2.31651 | 1776602 |
| 1.19859 | 765531 |
| 1.54737 | 2140923 |
| 2.02502 | 3556332 |
| 1.2668 | -742294 |
| 1.13197 | 1596853 |
| 0.793252 | 692447 |
| 1.03888 | 266495 |
| 1.34014 | 562169 |
| 2.53367 | 2580803 |
| 1.63525 | 1923345 |
| 1.21339 | 2584534 |
| 1.42084 | 874210 |
| 0.810837 | -405960 |
| 0.594715 | 1011779 |
| 2.7852 | 2988371 |
| 0.500819 | 938322 |
| 2.8397 | 1136404 |
| 1.42274 | 637815 |
| 1.05626 | 79550.3 |
| 0.354883 | 972721 |
| 0.974586 | 141364 |
| 1.52871 | 2229859 |
| 2.08687 | 1462689 |
| 1.97166 | 2579956 |
| 1.65799 | 2513230 |
| 1.39294 | 500699 |
| 1.1316 | 1094767 |
| 1.92577 | 1949394 |
| 0.95832 | 1575123 |
| 1.48675 | 1661731 |
| 2.2108 | 1955743 |
| 1.58831 | 2858427 |
| 1.06147 | 893806 |
| 2.177 | 2444571 |
| 1.51977 | 1281279 |
| 2.14145 | 2919531 |
| 1.05763 | 183947 |
| 2.00203 | 2013146 |
| 1.65279 | 2475664 |
| 1.82491 | 3770618 |
| 2.01605 | 2079000 |
| 2.39656 | 5946541 |
| 2.02742 | 179672 |
| 1.81055 | 3049584 |
| 0.431587 | 807468 |
| 2.04944 | 2833282 |
| 0.280918 | -1084887 |
| 1.68687 | 2467033 |
| 1.91189 | 1471098 |
| 0.995705 | 81765.4 |
| 1.27193 | 1773678 |
| 1.87144 | 540240 |
| 1.61505 | 1873391 |
| 2.41367 | 2172415 |
| 0.307966 | -1019928 |
| 1.60891 | 2067098 |
| 1.14839 | 1698300 |
| 1.10609 | 1709636 |
| 2.64436 | 1568017 |
| 0.948015 | 1537558 |
| 1.34344 | 225970 |
| 1.29558 | 2537199 |
| 1.49065 | 1566293 |
| 2.20068 | 3101187 |
| 1.33622 | 1423793 |
| 1.51134 | 2165382 |
| 1.12814 | 586553 |
| 2.14749 | 2109479 |
| 1.32712 | 554908 |
| 2.07982 | 1324985 |
| 1.42536 | 2001388 |
| 0.912379 | 2626703 |
| 0.626364 | 99868.3 |
| 2.18089 | 2850009 |
| 1.78624 | 3684501 |
| 2.13315 | 1364415 |
| 1.71011 | 1172891 |
| 2.10972 | 3164997 |
| 0.92988 | 1362330 |
| 1.71822 | 2055486 |
| 1.40991 | 3125294 |
| 1.85184 | 3023503 |
| 1.31765 | -32489.2 |
| 2.6353 | 3077738 |
| 1.60348 | 459427 |
| 1.32349 | 1955104 |
| 1.93241 | 1783914 |
| 1.90539 | 1128297 |
| 2.50215 | 743484 |
| 1.6185 | 2487857 |
| 1.08063 | 1275416 |
| 1.23185 | 793542 |
| 2.56902 | 1491892 |
| 1.71511 | 2315903 |
| 1.97084 | 3182973 |
| 0.778047 | 1353391 |
| 0.40596 | 709263 |
| 2.04158 | 1664629 |
| 0.950419 | 479662 |
| 1.37051 | 3326722 |
| 2.34484 | 2785118 |
| 0.623472 | 418856 |
| 1.7757 | 2324950 |
| 2.4363 | 2210857 |
| 3.11662 | 2626130 |
| 2.02085 | 3282035 |
| 2.02673 | 287044 |
| 1.80775 | 3661948 |
| 1.3144 | 890260 |
| 0.881765 | 202931 |
| 1.5657 | 1245509 |
| 3.32547 | 2794380 |
| 1.25023 | 1832106 |
| 0.783365 | 1337121 |
| 0.637741 | 639592 |
| 1.13945 | 1777806 |
| 0.827069 | 556689 |
| 3.02536 | 2475631 |
| 1.65012 | 1012467 |
| 2.84299 | 3890132 |

| Incremental Effectiveness (QALY) | SCE 5 |
| --- | --- |
| -1.65986 | 599309 |
| -1.00024 | -825831 |
| 0.0901077 | 787697 |
| 0.725137 | 1356383 |
| 0.745855 | -63572.4 |
| -0.797089 | -64971.5 |
| 0.871741 | 303417 |
| 0.00695495 | -302415 |
| 0.268002 | -267778 |
| 0.379929 | -229131 |
| 0.756175 | 2947258 |
| -0.362068 | 1462943 |
| 0.103596 | 1730114 |
| -0.467002 | 481228 |
| -0.275871 | 350360 |
| 0.110791 | 1135028 |
| 0.726618 | 1684249 |
| 0.835428 | 2386328 |
| -0.338672 | 390808 |
| 0.141727 | -248582 |
| -1.04074 | 1054362 |
| 0.403453 | 2936711 |
| -0.055748 | 2402048 |
| -0.666953 | -1237379 |
| 0.0961973 | 1150239 |
| 0.176548 | 24144.4 |
| 0.334126 | 1702276 |
| 0.198392 | -75202.2 |
| 0.531383 | 2622863 |
| 1.29772 | 1432420 |
| -0.90305 | -1595503 |
| 1.24139 | 719620 |
| 0.283049 | 1867291 |
| -0.023525 | 1175233 |
| -0.106422 | 897781 |
| -1.4353 | -3278317 |
| 0.968926 | 565358 |
| -0.545825 | 346776 |
| 0.0839454 | -317313 |
| -1.0051 | -1373617 |
| 0.834087 | -768327 |
| 0.0720105 | 1704067 |
| -0.312976 | -43423.6 |
| -0.657949 | -908657 |
| 1.09432 | 407245 |
| -0.214353 | 920597 |
| 0.305051 | 1399729 |
| 0.111769 | 2189724 |
| 0.0118402 | 113787 |
| 0.269103 | -226002 |
| 0.376633 | -509779 |
| -0.59282 | -282995 |
| 0.168011 | 374304 |
| -0.755218 | 278458 |
| -0.915736 | -401642 |
| 0.808267 | 2385798 |
| -0.500097 | 523646 |
| -0.117609 | 1298303 |
| 0.106017 | 186811 |
| -0.392691 | 1093957 |
| -0.229419 | -710630 |
| 0.30936 | 1152409 |
| -0.176285 | 465125 |
| -0.627034 | -284824 |
| 0.065848 | 3971772 |
| -0.010872 | 1283686 |
| -0.555642 | 86763.1 |
| -0.367148 | -612357 |
| 1.1774 | 1492479 |
| 0.421428 | 5700.01 |
| -0.662804 | -1016.71 |
| 0.195012 | 18070.2 |
| 0.0625106 | 220892 |
| 0.608124 | 1389088 |
| 0.248729 | 820872 |
| 0.0520759 | 87895 |
| 0.604569 | 1450066 |
| -1.19687 | -1301417 |
| -1.55529 | 182987 |
| 0.996035 | 864251 |
| -0.446206 | -191660 |
| -0.282362 | 216571 |
| 0.578265 | 2569200 |
| -0.44774 | -1746007 |
| -0.529223 | 512128 |
| -0.907528 | -229931 |
| -0.843848 | -1473256 |
| -0.339289 | -378556 |
| 1.02797 | 1411623 |
| 0.121204 | 491966 |
| -0.192524 | 1916766 |
| -0.0284925 | 138345 |
| -0.884107 | -1886851 |
| -1.01622 | -498964 |
| 1.27818 | 2158580 |
| -1.33125 | -644257 |
| 1.11403 | 52184.3 |
| -0.11079 | -439204 |
| -0.186648 | -670383 |
| -1.21826 | 132010 |
| -0.748857 | -1591044 |
| -0.0479211 | 884747 |
| 0.177602 | 426054 |
| 0.450921 | 1647396 |
| 0.127153 | 1409745 |
| -0.477089 | -540075 |
| -0.215469 | -396860 |
| 0.0251685 | 909167 |
| -1.01394 | 79901 |
| -0.113171 | 538361 |
| 0.47225 | 665803 |
| -0.142529 | 1793532 |
| -0.461147 | -85591.3 |
| 0.506196 | 1156687 |
| -0.0475554 | -311648 |
| 0.50187 | 1400472 |
| -0.809693 | -1581237 |
| 0.608515 | 1100930 |
| 0.384351 | 1559891 |
| 0.125399 | 2355260 |
| 0.365976 | 1283621 |
| 0.798861 | 5012382 |
| 0.428033 | -690182 |
| 0.145332 | 2008394 |
| -1.2945 | -518150 |
| 0.552337 | 1796322 |
| -1.45093 | -2541772 |
| -0.365549 | 675814 |
| 0.261351 | 322261 |
| -1.14286 | -1682510 |
| -0.39511 | 210570 |
| 0.362751 | -272117 |
| 0.131384 | 497749 |
| 1.19185 | 1176919 |
| -1.37472 | -2580539 |
| -0.0112902 | 1168552 |
| -0.479869 | 560374 |
| -0.617598 | 620592 |
| 1.4226 | 125860 |
| -0.545464 | 568814 |
| -0.549333 | -1440568 |
| -0.437081 | 935126 |
| -0.313444 | 610233 |
| 0.846564 | 2308496 |
| -0.391875 | -115066 |
| -0.0741092 | 848333 |
| -0.546505 | -564159 |
| 0.416321 | 1167130 |
| -0.261499 | -492782 |
| 0.59269 | 7651.17 |
| -0.22308 | 494592 |
| -0.346499 | 2152502 |
| -1.16534 | -1308349 |
| 0.59122 | 2317841 |
| 0.124497 | 1907246 |
| 0.539415 | -124687 |
| -0.288518 | 802828 |
| 0.077705 | 1478034 |
| -0.88006 | -58626.1 |
| 0.154615 | 645150 |
| 0.0643532 | 1991144 |
| 0.27199 | 1660796 |
| -0.484574 | -1283744 |
| 0.80357 | 1556212 |
| -0.383876 | -858749 |
| -0.0983553 | 848438 |
| -0.151219 | 295171 |
| 0.29526 | 153196 |
| 0.908843 | -659250 |
| 0.0714271 | 1296562 |
| -0.437174 | 364271 |
| -0.515042 | -695282 |
| 1.23309 | 658228 |
| -0.0640025 | 1516695 |
| 0.240818 | 2509604 |
| -0.923729 | -139415 |
| -1.46164 | -785901 |
| 0.438476 | 214569 |
| -0.996047 | -933524 |
| -0.25383 | 2170394 |
| 0.470176 | 1646307 |
| -0.883146 | -841706 |
| -0.00456534 | 1206074 |
| 0.824387 | 1075265 |
| 1.48485 | 1895161 |
| 0.027686 | 1966323 |
| 0.432211 | -554164 |
| 0.180904 | 2475092 |
| -0.584394 | -665437 |
| -0.69935 | -1177059 |
| 0.231778 | 570482 |
| 1.98234 | 2526367 |
| -0.725772 | 732277 |
| -1.1994 | -200640 |
| -1.28287 | -798594 |
| -0.588801 | 425576 |
| -0.924704 | -713079 |
| 1.36702 | 1206855 |
| 0.00696625 | -69008.8 |
| 1.37288 | 3285765 |

| Incremental Effectiveness (QALY) | Mean ICER |
| --- | --- |
| 8.21097 | 14167758 |
| 8.40889 | 14723351 |
| 9.44233 | 14548898 |
| 9.85541 | 15873981 |
| 8.21097 | 14696674 |

| Incremental Effectiveness (QALY) | WTP Threshold |
| --- | --- |
| 0 | 0 |
| 6 | 720000 |

Figure 9: Cost-effectiveness plane- Scenario approach (Societal perspective)

# E. **Data collection tools**

| Date of Interview \| \| \| Research Assistant Name \| \| \|; SECTION 1: PATIENT DEMOGRAPHICS \| \| \| \| \| \|; Patient OP/IP number \| \| \| \| Age \| \| Sex; Area of residence \| \| \| \| County of origin \| \|; Patient highest level of education Primary Secondary Tertiary No education \| \| \| \| \| \|; Marital Status Never married/ Single Married Widowed/ Separated/ Divorced \| \| \| \| \| \|; Occupation Employed Self-employed Unemployed Retired Student \| \| \| \| \| \|; Household Income per month Less than Kshs. 20,000 Kshs 20,001-50,000 Kshs 50,001-Kshs, 100,000 More than Kshs. 100,000 Other____ \| \| \| \| \| \|; How much does your household spend on essential items such as food, rent, utilities, school fees, healthcare (including health insurance), medicines, etc every month ____________ \| \| \| \| \| \|; Do you have health insurance? Yes No \| \| \| \| \| \|; Insurance cover No insurance Employer-based Privately-purchased health insurance NHIF/SHA Community-based health insurance Other ­­­­­­­­­­­­_____________________________ \| \| \| \| \| \|; How much do you spend on health insurance every month? __________ \| \| \| \| \| \|; SECTION 2: HEALTH-RELATED CHARACTERISTICS \| \| \| \| \| \|; Patient diagnosis \| \| \| \| \| \|; Comorbidities Diabetes Mellitus Hypertension Heart failure Others ________________________________ \| \| \| \| \| \|; Inpatient Outpatient \| \| \| \| \| \|; Current Kidney Replacement Therapy Haemodialysis Peritoneal Dialysis Kidney Transplant \| \| \| \| \| \|; Date of commencement of current KRT \| \| \| \| \| \|; Patient been on any other KRT Yes No \| \| \| \| \| \|; If yes when did they start and stop? \| \| \| \| \| \|; Reason for changing from previous to current therapy \| \| \| \| \| \|; SECTION 3: COSTS \| \| \| \| \| \|; 1. \| Consultation \| Fee per visit______ \| \| \| Number of times in the last three months_______ \|; 2. \| Diagnostics (Laboratory& Radiology) \| Type of service ________ & the fee_________ \| \| \| Number of times in the last three months_______ \|; 3. \| Surgical procedures \| Type of service ________ & the fee_________ \| \| \| Number of times in the last three months _______ \|; 4. \| Inpatient fee \| Bed charge per day _____ \| \| \| Length of stay ________ \|; 5. \| Medication \| Type of medication _________ Number of units ________ \| \| \| price per unit ___________ + dispensing fee \|; Direct Non-Medical Costs \| \| \| \| \| \|; 1. \| Transport \| i.Transport cost ________ Or ii. Distance to facility ___ cost per km _______ \| \| \| Number of visits in the last three months_______ \|; 2. \| Food \| Cost for meals per day \| \| \| Number of days of hospital visit per month________ \|; 3. \| Accommodation \| Cost per night \| \| \| Length of stay _______ \|; 4. \| Informal care \| Cost per day \| \| \| Number of days in the last three months _________ \|; Indirect Costs \| \| \| \| \| \|; 1. \| Patient-time loss \| Income per day \| \| \| Number of days in the last three months __________ \|; 2. \| Caregiver-time loss \| Income per day \| \| \| Number of days in the last three months __________ \| **APPENDIX 5- EUROQOL GROUP EQ-5D-5L& EQ-VAS QUESTIONNAIRE** | |
| --- | --- |
|  | |
| **First, I would like to ask you about MOBILITY. Would you say that:** | |
| You have no problems in walking? | ❑ |
| You have slight problems in walking? | ❑ |
| You have moderate problems in walking? | ❑ |
| You have severe problems in walking? | ❑ |
| You are unable to walk? | ❑ |
|  | |
|  | |
| **Next, I would like to ask you about SELF-CARE. Would you say that:** | |
| You have no problems washing or dressing yourself (putting on your own clothes)? | ❑ |
| You have slight problems washing or dressing yourself (putting on your own clothes)? | ❑ |
| You have moderate problems washing or dressing yourself (putting on your own clothes)? | ❑ |
| You have severe problems washing or dressing yourself (putting on your own clothes)? | ❑ |
| You are unable to wash or dress yourself (put on your own clothes)? | ❑ |
|  | |
|  | |
| **Next, I would like to ask you about USUAL ACTIVITIES (for example work, study, housework, family or leisure activities.) Would you say that:** | |
| You have no problems doing your usual activities? | ❑ |
| You have slight problems doing your usual activities? | ❑ |
| You have moderate problems doing your usual activities? | ❑ |
| You have severe problems doing your usual activities? | ❑ |
| You are unable to do your usual activities? | ❑ |
|  | |
|  | |
| **Next, I would like to ask you about PAIN OR DISCOMFORT. Would you say that:** | |
| You have no pain or discomfort? | ❑ |
| You have slight pain or discomfort? | ❑ |
| You have moderate pain or discomfort? | ❑ |
| You have severe pain or discomfort? | ❑ |
| You have extreme pain or discomfort? | ❑ |
|  | |
|  | |
| **Finally, I would like to ask you about WORRY (ANXIETY) OR DEPRESSION. Would you say that:** | |
| You are not worried or depressed? | ❑ |
| You are slightly worried or depressed? | ❑ |
| You are moderately worried or depressed? | ❑ |
| You are severely worried or depressed? | ❑ |
| You are extremely worried or depressed? | ❑ |

The best health you can imagine

**EQ-5D VAS**

- **Now, I would like to ask you to say how good or bad your health is TODAY.**

- **I would like you to picture in your mind a vertical line that is numbered from 0 to 100.**

(Note to interviewer: if interviewing face-to-face, please show the respondent the VAS line.)

- **100 at the top of the line means the best health you can imagine.**

**0 at the bottom of the line means the worst health you can imagine.**

- **I would now like you to tell me the point on this line where you would put your health TODAY.**

(Note to interviewer: mark the line at the point indicating the respondent’s health today. Now, please write the number you marked on the line in the box below.)

THE RESPONDENT’S HEALTH TODAY =

The worst health you can imagine
